# Printability and Performance Metrics of New-Generation Multifunctional PMMA/Antibacterial Blend Nanocomposites in MEX Additive Manufacturing

**DOI:** 10.3390/polym17030410

**Published:** 2025-02-04

**Authors:** Markos Petousis, Nektarios K. Nasikas, Vassilis Papadakis, Ioannis Valsamos, Katerina Gkagkanatsiou, Nikolaos Mountakis, Apostolos Argyros, Evgenia Dimitriou, Nikolaos Michailidis, Nectarios Vidakis

**Affiliations:** 1 Department of Mechanical Engineering, Hellenic Mediterranean University, 71410 Heraklion, Greece; markospetousis@hmu.gr (M.P.); valsamos@hmu.gr (I.V.); gkagka@hmu.gr (K.G.); mountakis@hmu.gr (N.M.); 2 Division of Mathematics and Engineering Sciences, Department of Military Sciences, Hellenic Army Academy, 16673 Attica, Greece; nasikas@sse.gr; 3 Institute of Electronic Structure and Laser of the Foundation for Research and Technology-Hellas (IESL-FORTH)—Hellas, N. Plastira 100 m, 70013 Heraklion, Greece; v.papadakis@uniwa.gr; 4 Department of Industrial Design and Production Engineering, University of West Attica, 12243 Athens, Greece; 5 Physical Metallurgy Laboratory, Mechanical Engineering Department, School of Engineering, Aristotle University of Thessaloniki, 54124 Thessaloniki, Greece; evgeniaod@auth.gr (E.D.); nmichail@auth.gr (N.M.); 6 Centre for Research & Development of Advanced Materials (CERDAM), Centre for Interdisciplinary Research and Innovation, Balkan Centre, Building B’, 10th km Thessaloniki-Thermi Road, 57001 Thessaloniki, Greece

**Keywords:** poly(methyl methacrylate) (PMMA), material extrusion (MEX), additive manufacturing, nanocomposites, mechanical characterization, *Staphylococcus aureus*, *Escherichia coli*

## Abstract

Poly(methyl methacrylate) (PMMA) is a thermoplastic widely utilized in civilian-, defense-, and medicine-related applications. Therefore, inducing antibacterial properties is an additional asset when infection control is prioritized. To counter this, PMMA was mixed, for the first time, with antibacterial agents (antibacterial blend nanopowder, AP) to curb bacterial proliferation and therefore reduce the chances of infection. The reinforcing efficacy of the blend in PMMA was also assessed. Nanocomposites were developed with various nanopowder concentrations for 3D printing material extrusion (MEX). PMMA/AP nanocomposites were evaluated for their mechanical and rheological properties, thermal stability, morphological, structural, and chemical characteristics, and bacterial resistance (against *Staphylococcus aureus* and *Escherichia coli* (*E. Coli*) using the well diffusion method). The effect on quality metrics, such as the geometrical accuracy and pores of the 3D-printed structure was examined with micro-computed tomography. The modified PMMA had improved properties, such as increased tensile (~20% increase at 2 wt.%) and flexural strength (~10.8% at 4 wt.%), while also having strong antibacterial properties against Staphylococcus aureus and mild antibacterial properties against *E. Coli*. Such improvements add to the expanding portfolio of biomaterials, such as their use in the demanding defense sector and the medical field.

## 1. Introduction

Novel material synthesis, along with novel manufacturing techniques, has always been the subject of extensive research in the quest for low-cost and easy-to-produce materials for various applications [1,2,3]. In this context, the traditional manufacturing techniques of forging, casting, or chiseling have given way to novel manufacturing techniques, such as additive manufacturing (AM). As the name implies, AM is a technique where several layers of a material are added, usually one on top of the other, in order to produce three-dimensional structures. The latter is also the reason why AM is frequently termed three-dimensional printing or just 3D printing (3DP) [4,5,6]. Probably the most common materials used in 3DP are polymers; this is mostly due to their interesting physicochemical and mechanical properties, which have led to their extensive use in numerous applications, from those of everyday life to more exotic ones [7,8,9,10]. AM has provided the ability to manufacture complex geometries in a way that sometimes was not even possible before without having to spare large quantities of material or using some kind of welding technique that could compromise durability and mechanical strength [11,12]. Recently, AM has taken over some very important fields, such as the defense and security sector, providing technical solutions that did not exist in the past [13,14,15].

In this context, AM has provided a very intriguing framework for various novel approaches in the manufacturing process of the different materials being utilized as filaments in order to produce the final 3DP products. Recent works in this respect have demonstrated the ability to fabricate polymeric filaments bearing various additives. Titanium Nitride, TiN [16]; Antimony Tin Oxide, ATO [17,18]; Silica glass [19]; Titanium Carbide, TiC [20]; and Silicon Nitride [21] are some of these additives.

The above-mentioned additives, in most cases, introduce specific multi-functionalities when incorporated into a specific matrix material. It has been shown that this can improve the mechanical properties of the final composite material [18,19,20,21] and also provide novel functionalities such as antimicrobial and biocidal properties [22]. These multi-functionalities have proven to be very important in the field of defense technology, as they can provide a reliable alternative manufacturing method that is cost-effective and quicker than traditional manufacturing methods and also enhances the performance and importance of these materials in extreme and demanding environments [22,23,24].

With the term demanding environments, we describe, for example, the need for enhanced mechanical properties that can endure, for instance, ballistic loading or withstand extreme heat after blasting or explosive detonation. These environments can be frequently addressed in armed conflicts or, in general, in defense and security-related activities.

The 3DP manufacturing technique has increasingly been utilized in defense and security-related activities and applications providing various innovative solutions and products [25,26,27]. The focus nowadays is on the nature of the multifunctional materials that are being used as filaments for the fabrication of the 3DP specimens. In this respect, multifunctionality usually comes from the additive material that is being incorporated into the polymer matrix. In this direction, we have seen the use of various additives such as ceramics [28,29,30,31] or other materials characterized as environmentally friendly materials [32,33,34,35,36].

Despite the extreme environments that are frequently addressed in the defense and security domain, another important aspect is health-related applications. Multifunctional materials play a key role in this domain, as they can be utilized in various health-related applications utilizing 3DP [37,38,39,40]. In this respect, the development of multifunctional materials that can possess bioactive properties, exhibit prolonged service life, foster cell growth, and show increased biocompatibility, can serve various needs that frequently arise in the defense and security domain, such as for medical emergencies, wound healing, or injury recovery. For instance, in today’s battlefields, there has been a significant shift towards the use of non-lethal weapons (NLWs) that have the ability to impose severe injuries rather than direct casualties. The aim for this is twofold. On one hand, a severely injured individual calls for emergency response and care, occupying human capital, while on the other hand, it creates psychological distress to those around the wounded individual [41,42]. The need for and importance of polymeric composites with antibacterial properties has been stated often in the literature, while the research in the field is also extensive. Various types of fillers (carbon-based or other) and polymeric composite preparation methods have been introduced, often not considering their use in AM methods [43,44,45,46,47].

Another interesting characteristic of today’s battlefields is the so-called “urbanization of warfare” [48]. In such an urban environment, those operating in it might likely find themselves not only under direct enemy fire but also in danger of debris originating from artillery shells firing at buildings. Wounds from large debris can cause severe life-threatening and non-life-threatening injuries that need an emergency response [49,50]. Therefore, it is extremely important to investigate the possibilities that 3DP methods can provide for the fabrication of various materials and components that can be very useful on the battlefield.

A large variety of biocompatible and biomedical-grade materials are widely available, metals and metal alloys, ceramics, bioactive glasses, and amorphous polymers being the most notable [51,52]. The combination of various bioactive and biocompatible materials with different 3DP techniques has shown to be quite effective, especially when custom-made solutions and materials are needed. This is also very important in defense-related applications where the rapid fabrication of various parts is needed, and these materials must be fabricated on-site during military operations or drills [53,54,55]. In this context, various composite materials have been reported to exhibit bioactivity, such as the composites containing silver nanoparticles (AgNPs) that have shown extensive antimicrobial activity against various pathogens [56,57,58,59].

Poly(methyl methacrylate) or PMMA is a synthetic polymer that originates from methyl methacrylate and mostly exhibits an amorphous structure. It is a widely used engineering thermoplastic and transparent in most cases, thus it is termed acrylic glass or, more commonly, Plexiglas [60,61,62]. This has led to the widespread use of PMMA sheets as a shatterproof alternative to ordinary glass. PMMA is also known to swell and dissolve in most organic solvents, but when compared to other plastics, like polyethylene or polystyrene, it exhibits superior environmental stability, thus making PMMA a polymer of choice when dealing with outdoor applications. In most applications, PMMA will not shatter but rather break into large pieces; however, it scratches more easily than ordinary glass.

PMMA is also known to exhibit a very good degree of biocompatibility, thus making it a very commonly used material in medicine and dentistry. It has been used as an intraocular lens in refractive surgery [63], in pharmaceutical “lab-on-a-chip” applications [64], in 3DP fillers in cranioplasty [65], in cosmetic and plastic surgery [66], and in dentistry [67]. This highlights the importance of PMMA as a material that can find important applications in various medical fields, such as in ophthalmology in film form (with its antibacterial properties tested against *Escherichia coli*) [68], among others. Aiming to improve its performance in medical applications, biocidal properties were induced into the thermoplastic using graphene and silver nanoparticles as additives [69]. PMMA is used as a cement for bone recovery and silver [70] or gold [71] particles have been used in such applications to add antibacterial capabilities to the PMMA composites. Other approaches for inducing biocidal properties include the use of TiO_2_ coatings [72] and the introduction of nitride [73] or zinc dimethacrylate [74] additives. Composites can developed using a process other than the one used in the current research and the additives can also differ, using ones not intended for use in material extrusion (MEX) 3D printing. Still, such studies highlight the interest in using PMMA with biocidal properties in various applications.

In 3D printing, although the performance of PMMA has been investigated, no such studies have been found to date in the bibliography. Still, the reported findings can safely recommend PMMA as an excellent material for 3DP. The energy required to build PMMA parts with the MEX method has been reported, while optimization conducted for the 3D printing settings’ values to achieve higher tensile strength [75]. As in the other manufacturing methods, 3D-printed PMMA has been employed in reflector mirrors [76] and optical applications [77]. In the medical field, it has been tested for use in dental applications [78], such as in prosthodontics [79] and in implants [80,81].

PMMA is used in various types of defense applications, from armor [82] to aircraft windows (civilian or military) [83]. In the latter, its resistance to weathering is a key parameter that enables its use; it is used in military applications for solar blinds [84]. Therefore, its resistance against impact loading and explosives has been investigated under various configurations and scenarios, while modeling tools have been applied as well [82,85,86,87,88]. The resistance against water-jet impact is an example of the studies conducted for aeronautical applications [89]. Its ballistic performance has also been evaluated [90], along with its resistance against chemical warfare agents in defense [91].

Although PMMA and other polymers have been extensively studied for their mechanical and physicochemical properties, very little attention has been given to PMMA composites with antibacterial additives used in the fabrication of filaments for material extrusion (MEX) 3DP. The need for multifunctional materials that can be used for 3DP and at the same time exhibit antibacterial properties is extremely important for the defense and security domain, where there is a growing need for implants, medical scaffolds, and personalized military equipment.

In the above direction, we have prepared in this work a series of PMMA composites bearing different loads of antibacterial nanoparticles, aiming to create filaments suitable for 3DP through the MEX method. The investigation protocol included several experimental and characterization techniques, such as Energy Dispersive X-ray spectroscopy (EDS), Scanning Electron Microscopy (SEM), mechanical properties assessment for the filaments and the 3DP examples, as well as melt flow rate (MFR) and viscosity, followed by evaluating their antibacterial performance.

The composites exhibited antibacterial behavior against the two assessed bacteria, namely gram-positive Staphylococcus aureus (*S. aureus*) and gram-negative *Escherichia coli* (*E. coli*). The efficacy was better against the *S. aureus* bacterium. Nanocomposites with such qualities can be utilized in industrial fields requiring robust mechanical performance and biocidal properties from the AM parts, such as in the defense and security sector.

To our knowledge, this is the first detailed work investigating PMMA nanocomposites exhibiting antibacterial properties while enhancing their mechanical performance within the context of the MEX 3D printing technology. The gap in the literature this research covers is the introduction of multifunctional nanocomposites for the material extrusion 3D printing method utilizing PMMA as the matrix material. The proposed nanocomposites aim to expand the use of the popular PMMA polymer in 3D printing in applications requiring robust performance from the material and antibacterial capabilities at the same time. Such nanocomposites featuring the PMMA polymer and antibacterial blends have not been presented so far for the MEX method, according to the conducted literature review. The increase in the popularity of 3D printing in applications has established a constant need for materials with enhanced properties, while the combination of these two material characteristics (strength and antibacterial properties) is sought-after and has merit in applications in the medical, culinary, and defense fields, in which PMMA is already widely used due to its characteristics. Our findings pave the way for relevant applications that can enhance the efficiency and health levels of the troops operating in combat zones.

## 2. Materials and Methods

**Figure 1:**
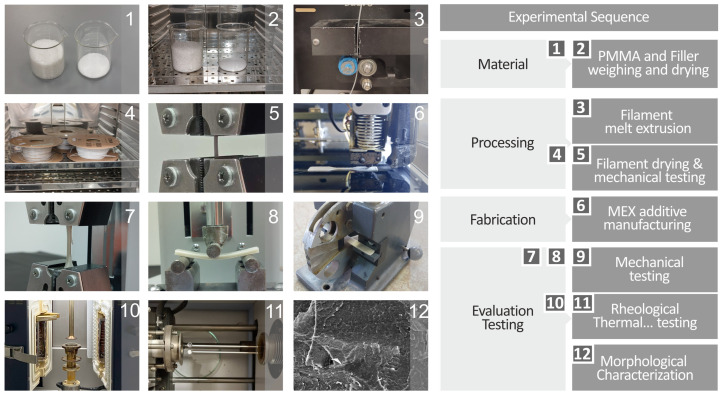
The preparation protocol followed in this work depicts (**1**) the preparation of the raw materials, (**2**) their subsequent drying, (**3**) the filament extrusion and drying process (**4**), (**5**) filament quality evaluation and mechanical tests, and (**6**) the 3D manufacturing of the samples utilizing the MEX method and (**7**) the subsequent quality examination, (**8**,**9**) their mechanical properties investigation (**10**,**11**), their rheological and thermal properties testing, and finally (**12**) their morphological characterization.

Figure 1 depicts the main protocol for the experimental sequence used for preparing the raw materials, the processing for filament extrusion and the subsequent mechanical testing, the MEX additive manufacturing of the various specimens, and finally the evaluation and testing regarding the mechanical, rheological, and morphological characteristics of the various prepared specimens. The initial steps involved careful weighing of the desired raw materials’ quantities. The raw materials were dried in an oven at ~60 °C for ~8 h in order to remove any absorbed moisture. The starting raw materials were mechanically mixed in the appropriate quantities and formed homogeneous mixtures for each batch of the desired final composites. The next step was to feed the prepared composites into the extruder, which in turn produced the various filaments. The drying process was also applied to all the prepared filaments, which were also inspected for their overall quality and mechanical properties, aiming to ensure that the as-prepared filaments were suitable for 3DP. The final 3DP composite specimens were also thoroughly tested for their mechanical properties, morphological and rheological characteristics, and overall antimicrobial nature.

### 2.1. PMMA and Antibacterial Nanopowder Materials

In this work, we utilized medical-grade PMMA resin purchased from Guangzhou Chozen Technology Co., Ltd. (Guangdong, China) in granule form and antibacterial nanopowder purchased from Nanografi (Ankara, Turkey). The antibacterial nanopowder blend, according to the datasheet provided by the manufacturer, had a silver (Ag) content of 3.45%, ZrO_2_ 46.41%, P_2_O_5_ 43.52%, Y_2_O_3_ 1.3%, N_2_O 1.92%, Al_2_O_3_ 0.39%, TiO_2_ 0.22%, and HfO_2_ 1.37%. Its bulk density is 0.53 g/mL, and it has an average particle size of 100 nm. These materials were appropriately weighed and thoroughly mixed in order to create a series of batches that were used as the precursors for the final composite materials.

Scanning Electron Microscopy (SEM), along with Energy-dispersive X-ray spectroscopy (EDS), were utilized to investigate the morphology and chemical configuration of the end products and were conducted using a Jeol JSM-IT700HR field-emission SEM (Jeol Ltd., Tokyo, Japan) under high vacuum at 20 kV, with samples being gold-sputtered.

### 2.2. PMMA Nanocomposites and Corresponding Filaments Preparation

Originally, the PMMA that was used as the matrix material was mixed with appropriate quantities of the antibacterial nanopowder in order to prepare the starting batches. The next step was the preparation of the corresponding filaments that have a diameter of 1.75 mm. For control purposes, a PMMA pure filament was also prepared. In this regard, a 3devo Composer 450 single screw extruder acquired by 3devo B.V., NL was used. After preliminary testing and consulting the literature, the appropriate temperatures for extrusion were set [92]. All filaments were produced (extruded) under identical settings to enable accurate comparison.

As stated above, the appropriate batches for all composites were prepared and placed in a high-power blender to ensure homogeneity. The batches were subsequently dried in an oven before being fed into the 3devo extruder that will produce the corresponding filaments. The final nanocomposites that were synthesized were PMMA pure and a series of PMMAs with an increasing content of antibacterial nanopowder, in wt.%, as follows. PMMA—2 wt.% antibacterial nanopowder, PMMA—4 wt.%, PMMA—6 wt.%, PMMA—8 wt.%, PMMA—10 wt.%, and PMMA—12 wt.%. The whole process of filament preparation was closely monitored to ensure defect-free filaments and also to account for the consistency of the process. To determine the loadings to be studied, the filler content gradually increased and samples were tested for their strength. When the mechanical performance started to decline, the process was terminated. The saturation of the filler in the matrix reduces the strength of the parts [93,94]. Herein, saturating the composites was not within the scope of the research. The aim was to improve mechanical performance while inducing biocidal properties to the nanocomposites, finding a balance between these two technological aspects.

### 2.3. 3DP of the PMMA—Antibacterial Nanocomposites

For executing the broad experimental protocol, we manufactured several 3DP specimens that were manufactured by utilizing a suitable 3D printing machine (in this case the model Funmat—HT was selected. It was purchased from a company named Intamsys, established in Shanghai, China). All specimens, namely the PMMA pure and the PMMA nanocomposites bearing different loadings of the antibacterial nanopowder, were 3DP utilizing an identical protocol for printing in order to ensure consistency and reproducibility.

### 2.4. Spectroscopic and Thermophysical Characterization of the PMMA—Antibacterial Nanocomposites

The powerful and non-destructive tool of Raman spectroscopy was utilized in order to investigate the various structural characteristics of the prepared nanocomposites. For this purpose, we utilized a micro-Raman setup from Labram HR-Horiba Scientific. The method is more analytically presented in the Supplementary Materials.

The thermophysical characteristics of the prepared samples were investigated by thermogravimetric (TGA) analysis and differential scanning calorimetry (DSC), aiming to elucidate the influence of the introduction of antibacterial nanoparticles into the PMMA. The TGA instrument used for the above-mentioned analysis was a Diamond Perkin Elmer calorimeter purchased from the Perkin-Elmer company, established in Massachusetts, United States. The temperature course used for the analysis was set between 40 and 300 °C. The step for the temperature increase was adjusted to 10 °C/min.

To investigate the thermal characteristics regarding the absorption of thermal energy of the polymer blends created and to extract important thermal metrics such as glass transition temperature, a Differential Scanning Calorimetry device was employed. To be more specific, an apparatus model named DSC 25 from the TA Instruments company, established in Delaware, United States was used. The device featured a Refrigerated Cooling System RCS 90, which allows for accurate temperature control in the range from −90 to 725 °C. The heating and cooling cycle used to evaluate the thermal behavior of the materials tested had a rate of 15 °C per minute and ranged from 25 to 280 °C. All measurements were conducted in an inert atmosphere using high-purity nitrogen gas.

### 2.5. Rheology Analysis of the PMMA—Antibacterial Nanocomposites

A characteristic that is very important when investigating the parameters that influence the 3DP process is the detailed knowledge of the rheological properties of the used filaments. In this regard, we analyzed the rheological properties of all the prepared filaments by utilizing a DHR-20 Discovery Hybrid Rotational Rheometer acquired from the company TA Instruments, established in Delaware, USA. The whole experimental system for the rheology property measurements involved an environmental test chamber equipped with parallel plate geometry, with dimensions regarding the diameter of the plates and the gap between the plates of 25 mm and 1 mm, respectively. To precisely control the temperature at which the experiments were conducted, the environmental test chamber was set to keep a constant temperature of 240 °C, which is also the printing temperature of the materials developed in this research.

The aim for measuring the rheological properties was to assess the possible deformation occurring in the examples under investigation when an external force is applied upon them, while the samples have transformed into their liquid phase. The transformation of the samples investigated from their solid-state phase to the liquid phase was ensured by exceeding their melting temperature (T_m_), while taking special care not to exceed the T_m_ substantially, eliminating this way the possibility of decomposing the materials under investigation. For every measurement point, we took 10 s to acquire the necessary values, ensuring that no decomposition effects would occur. The American Society for Testing and Materials (ASTM) standard D1238-10 for melt flow rate (MFR) was utilized for carrying out all relevant measurements. The temperature used during the MFR experiments was 230 °C, and the weight used was 3.8 kg.

### 2.6. Mechanical Response of the PMMA—Antibacterial Nanocomposites

The mechanical behavior of the PMMA-antibacterial nanocompounds was assessed by employing an MX2 device (Imada Inc., Northbrook, IL, USA). In order to ensure the consistency and reproducibility of the samples, we used five (5) different samples from each different material prepared for our measurements. The analysis was performed under typical ambient conditions, and the ASTM D638-02a international standard was employed for the tensile experiments, with a speed set at 10 mm/min. Examples were 3.2 mm thick and V-type.

### 2.7. Evaluation of the Morphological Characteristics and the Chemical Composition of the PMMA—Antibacterial Nanocomposites

For all the specimens prepared in this research, we examined their morphological characteristics and analyzed their chemical composition. We utilized the lateral surfaces and the surfaces of the samples that failed and broke during the tensile tests of the 3DP samples. The analysis was performed through SEM, aiming also to determine the overall quality and morphology of the prepared samples. Finally, the chemical analysis of the samples was performed through EDS on a JEOL field emission JSM-IT700HR device. The investigated samples we previously gold-sputtered, and the corresponding images were captured in high-vacuum mode set at 20 kV.

### 2.8. Quality Metrics Evaluation of the PMMA—Antibacterial Nanocomposites

The powerful tool of μ-computed high-resolution tomography (μ-CT-HD) scanning was utilized in order to examine the possible variation in the nominal dimensions between the computed-assisted designed geometrical and dimensional characteristics (60L resolution) of the various samples and the actual 3DP ones (Tomoscope HV Compact 225 kV Micro Focus, Werth Messtechnik GmbH, Giessen, Germany). The same method was used for the determination of the voids and porosity in the 3D printing structure (16L resolution). The corresponding data originating from the CT-voxel data set were processed by the software named VG Studio MAX 2.2 (Volume Graphics GmbH, Heidelberg, Germany).

### 2.9. Evaluation of the Biocidal Effect of the PMMA—Antibacterial Nanocomposites

The evaluation of the antibacterial action of the various PMMA—antibacterial nanocomposites was carried out by employing the method of agar well diffusion [95]. The setup and conditions have been described in detail in previous works [96,97]. The bacteria chosen for testing were gram-negative *E. coli* and gram-positive *Staphylococcus aureus* (*S. Aureus*). These bacteria were placed in suitable Petri dishes to be cultivated. The Petri dishes had a diameter of 85.0 mm [98,99] and contained the compatible cultivation nutrient agar for *E. Coli* (C 010066 Chapman) and *S. Aureus* (MC2, C 010068). The final solutions were prepared by utilizing the McFarland 0.5 process [100]. Subsequently, the 3DP samples were inserted in the petri dishes and their antibacterial performance was monitored and evaluated. The formation of an inhibition zone (IZ) in the Petri dishes was closely monitored through an optical microscope, following an incubation sequence at a steady temperature of 37 °C for 24 h. Aiming to ensure the consistency of our IZ determination protocol, we prepared five different specimens and recorded the mean IZ size and its deviation for the two pathogens tested. The estimation of the mean IZ size for each sample was determined by measuring six (6) different diameters at 60° angle increments and calculating the mean value, assuming a cyclic geometry. In this regard, the inner circle area—representing the sample—was subtracted from the greater IZ circle.

## 3. Results

### 3.1. Morphological and Chemical Assessment of the Antibacterial Nanopowder

**Figure 2:**
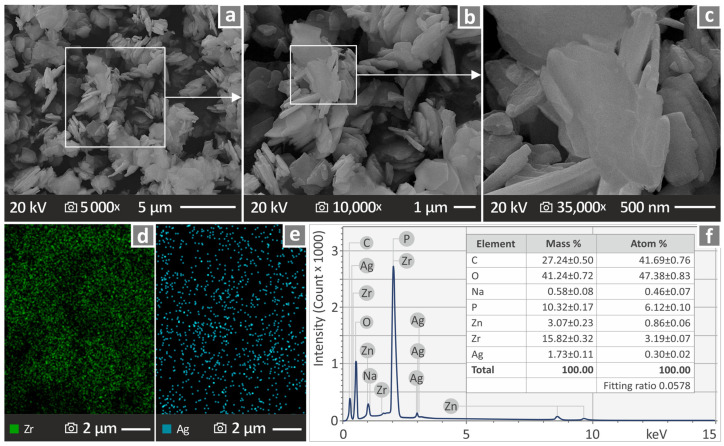
(**a**) SEM image of the antibacterial nanopowder at 5000×, (**b**) 10,000×, and (**c**) 35,000× magnification, (**d**) EDS mapping of Zr, (**e**) EDS mapping of Ag, and (**f**) EDS chemical composition of the antibacterial nanopowder.

Various SEM illustrations captured from the antibacterial nanopowder in three different magnifications and subsequent EDS analysis are shown in Figure 2a–f. Figure 2a shows a representative SEM image captured at 5000× magnification. The marked area in Figure 2a corresponds to the SEM image at 10,000× magnification, shown in Figure 2b. In the same sense, the marked area in Figure 2b corresponds to the SEM image at 35,000× magnification, shown in Figure 2c. Figure 2d,e show the dispersion of Zr and Ag through EDS mapping on the sample. Finally, Figure 2f shows an EDS graph of the elemental analysis performed on the antibacterial nanopowder. The particles’ nanoscale was found, along with their composition. In the SEM images, particle clustering is presented, owing to the process for acquiring the SEM images, as the antibacterial blend nanoparticles were applied on a carbon tape adhered to laboratory glass. It should be noted that the nanoparticles appear agglomerated, as they were placed in adhesive carbon tape fitted to a laboratory glass in order to acquire their SEM images.

### 3.2. Vibrational Spectroscopy Investigation of the PMMA—Antibacterial Nanocomposites

**Figure 3:**
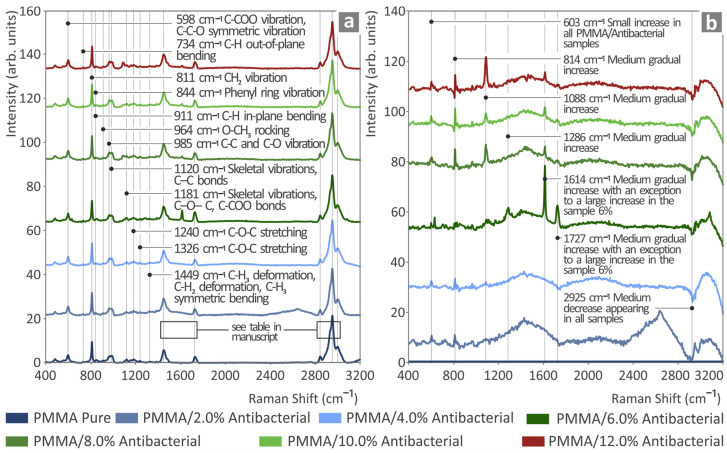
(**a**) Respective Raman spectroscopy findings from the unfilled PMMA and PMMA/2.0% antibacterial, PMMA/4%, PMMA/6%, PMMA/8%, PMMA/10%, and PMMA/12%, respectively. (**b**) Raman spectra of the PMMA—antibacterial nanocomposites after subtracting the PMMA pure Raman spectra, shown in the bottom of subfigure (**a**).

Vibrational spectroscopy, namely Raman spectroscopy, was employed in order to reveal, in detail, the structural characteristics of the various 3DP PMMA—antibacterial nanocomposites. The corresponding Raman spectral signatures originating from PMMA pure and the various loadings of PMMA—antibacterial nanocomposites are shown in Figure 3a. Figure 3b shows only the PMMA—antibacterial nanocomposites spectra after subtraction of the PMMA pure Raman spectrum. In Figure 3a, when increasing the antibacterial content, no major spectral alterations are observed, implying no major structural changes occurring in the nanocomposites; however, after subtracting the PMMA pure spectral signature, we observe the following alterations. The findings are documented in the literature [101,102,103,104,105,106,107,108] and presented in the Supplementary Materials in a table form.

The broad spectral envelope stretching from 1200 to 1700 cm^−1^ seems to be lowering in intensity, while several new and intense peaks appear for the PMMA/6% sample and samples with higher antibacterial nanopowder contents. This implies that some structural changes are observed in the PMMA—antibacterial nanocomposite as the incorporation of the nanomaterials takes place into to polymer matrix. The PMMA/6% nanocomposite also shows a sharp increase in the intensity of a newly appearing sharp peak at 1614 cm^−1^, which, despite the fact that it lowers in intensity, does not disappear as the antibacterial nanomaterial content increases. Other observed peaks also show similar behavior, with small to medium increases with increasing antibacterial content. These peaks are located at 603 cm^−1^, 814 cm^−1^, 1068 cm^−1^, 1286 cm^−1^, 1614 cm^−1^, 1727 cm^−1^, and 2925 cm^−1^, respectively. The addition of the antibacterial nanopowder in the PMMA modified the Raman spectra. The changes are listed in Table 1, complemented with the observations.

### 3.3. Rheometry and Melt Flow Rate Characteristics of the PMMA—Antibacterial Nanocomposites

**Figure 4:**
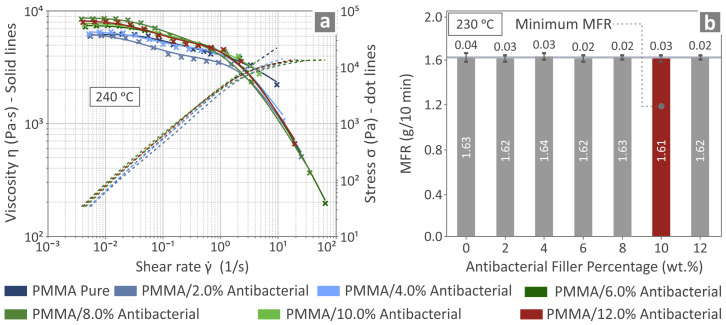
(**a**) Viscosity of the various PMMA pure and nanocomposites prepared. (**b**) MFR results from the PMMA pure and prepared nanocomposites.

As seen in Figure 4a, which shows the results from the rotational rheometry experiments, a gradual reduction in viscosity (η) was observed as the shear rates increased, showcasing a shear thinning behavior, as expected for the polymeric nature of the materials developed. Generally, the introduction of the antibacterial agent in the dosages described above has a minor effect on the viscosity of the materials developed. The same can be observed from the MFR experiments, where the MFR values showed a negligible change in regard to the incorporated additive percentage. The observations described above can be seen in Figure 4b.

### 3.4. Thermochemical Investigation of the PMMA—Antibacterial Nanocomposites

**Figure 5:**
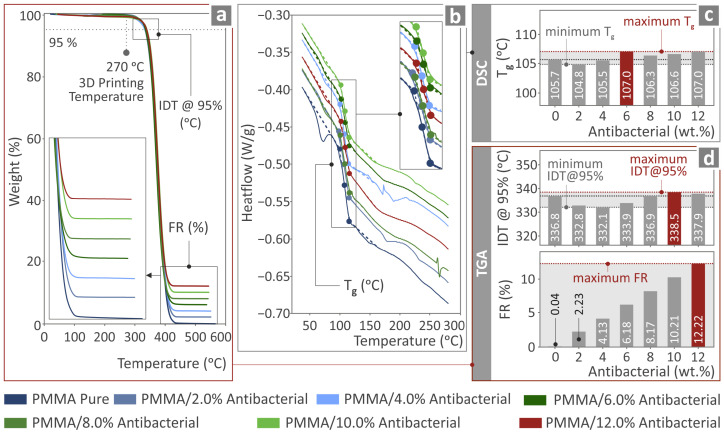
(**a**) TGA graph of the various PMMA pure and antibacterial nanocomposites. (**b**) DSC graphs for the determination of the T_g_ behavior with increasing antibacterial content. (**c**) The variation of the T_g_ with varying antibacterial content. (**d**) TGA-derived variation of the IDT and FR.

The TGA results (Figure 5a) depict no notable changes in the response to temperature of the PMMA due to the addition of the antibacterial blend nanoparticles. The temperature at which the acute degradation of the nanocomposites begins was raised by about 2 °C in the composite with 10 wt.% filler content. The residuals agree with the content of the filler in the nanocompounds. Additionally, it was verified that the temperatures applied for the extrusion of the filament and the 3D printing of the examples were lower than the temperature at which the acute degradation of the nanocomposites begins. Therefore, no degradation occurred in the nanocompounds that would negatively affect the mechanical properties of the 3D-printed nanocomposite parts.

From the DSC results shown in Figure 5, it can be observed that the produced materials presented only a characteristic change in the heat flow rate in the region near 106 °C, which is the glass transition temperature T_g_. The T_g_ depicted a fluctuation of less than 2%, denoting that the addition of antibacterial nanopowder into the matrix had an insignificant impact on the thermal properties of the created materials.

### 3.5. Investigation of the Thermomechanical Properties and Behavior of the PMMA—Antibacterial Nanocomposites

**Figure 6:**
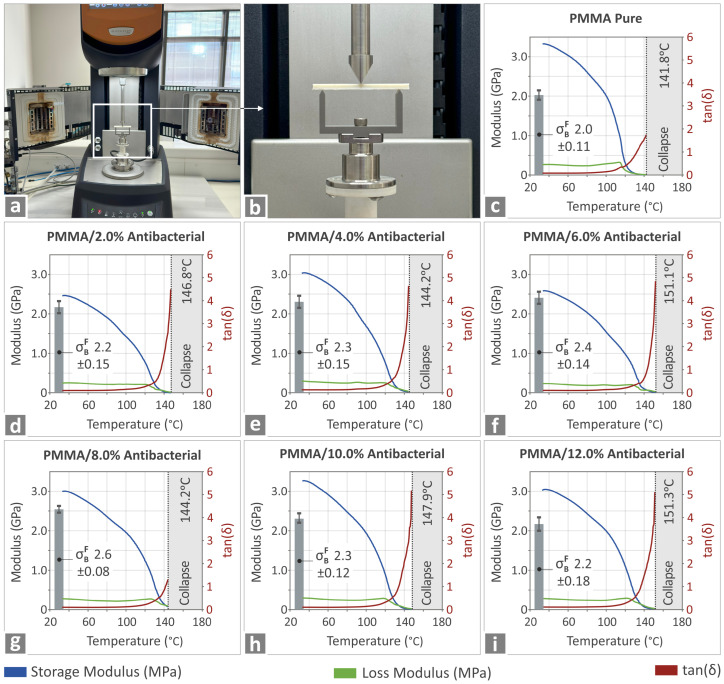
(**a**) Placing the sample for the investigation of the thermomechanical properties of the various PMMA—antibacterial nanocomposites. (**b**) Enhanced image of the specimen shown in the box in (**a**) for the three-point bending test. (**c**–**i**) Various graphs regarding the mechanical characteristics of the corresponding PMMA pure and PMMA—antibacterial nanocomposites.

Figure 6 shows the experimental setup and the corresponding results regarding the elucidation of the mechanical properties of the various PMMA—antibacterial nanocomposites manufactured via 3DP. All specimens were thoroughly investigated for their thermomechanical properties for various temperatures in the range from room temperature to 151.3 °C. The storage and loss modulus, along with tanδ, were simultaneously recorded, and the results are shown in Figure 6c–i. No major differences were recorded due to the incorporation of the antibacterial nanoparticles, implying that the thermomechanical properties are not significantly negatively affected when compared to the PMMA pure and in fact seem to be even enhanced to a small extent.

### 3.6. Quality Assurance of the Prepared PMMA—Antibacterial Nanocomposites Produced Filaments

**Figure 7:**
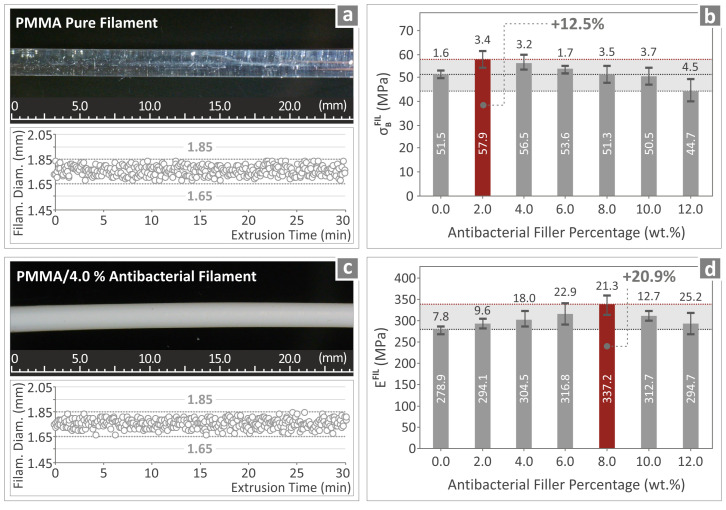
(**a**) Filament diameter monitoring and overall quality inspection for the PMMA pure and (**c**) PMMA/4.0% antibacterial filament. (**b**) Tensile strength and (**d**) Young’s modulus for all filaments prepared.

Aiming to ensure the high quality of the produced filaments, which correspond to the various PMMA—antibacterial nanocomposites studied herein, we systematically investigated their mechanical properties and overall characteristics. The corresponding results are shown in Figure 7a–d. The overall surfaces of the produced filaments for all composite materials appear to be smooth and free of any cracks or problematic roughness. The diameters of the produced filaments seemed to remain relatively constant, varying only from 1.65 to 1.85 mm, thus confirming the reproducibility and overall consistency of the produced filaments. Analogous results originating from the tensile tests of the produced filaments are shown in Figure 7b and Figure 7d, respectively. Figure 7b shows the tensile strength, while Figure 7d shows the tensile modulus of elasticity. For the tensile strength, the PMMA/2% antibacterial nanocomposite shows the best performance, with an increase of 12.5% when compared to the PMMA pure filament. For the tensile modulus of elasticity, the best performance is shown for the PMMA/6 wt.% antibacterial nanocomposite, with an increase of 20.9% compared to the PMMA pure filament.

### 3.7. Evaluating the Mechanical Response of the PMMA—Antibacterial Nanocompounds

**Figure 8:**
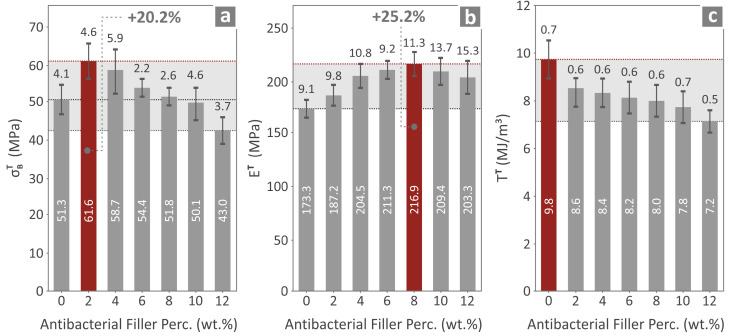
(**a**) Tensile strength, (**b**) tensile modulus of elasticity, and (**c**) tensile toughness of the various 3DP materials prepared.

**Figure 9:**
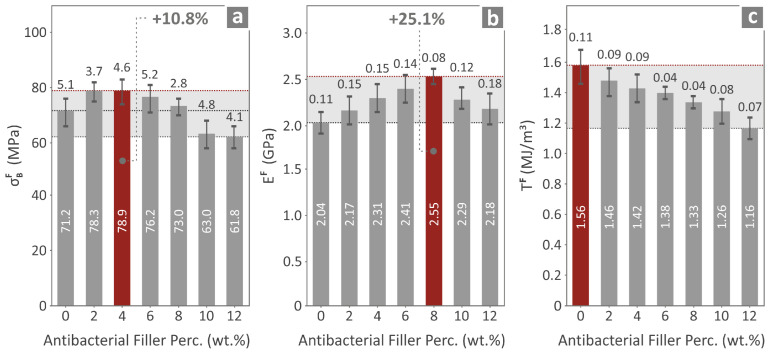
(**a**) Flexural strength, (**b**) flexural modulus of elasticity, and (**c**) flexural toughness of the various 3DP materials prepared.

**Figure 10:**
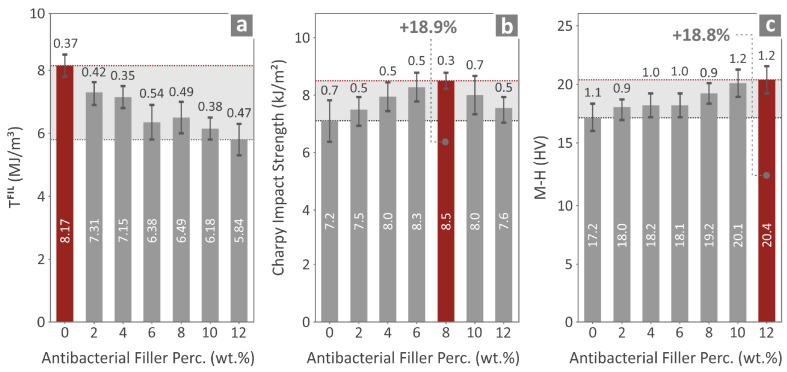
(**a**) Filament toughness, (**b**) impact strength (Charpy), and (**c**) Vickers microhardness of the various 3DP materials prepared.

In an effort to evaluate the mechanical properties of the prepared nanocomposites, a series of extensive mechanical tests were undertaken, and the relevant results are presented in Figure 8, Figure 9 and Figure 10. This comprehensive approach provides detailed knowledge of the mechanical properties of the various 3DP PMMA nanocomposites and reveals very interesting information. Figure 8a–c shows mean and deviation values for the tensile strength, Young’s modulus, and tensile toughness for all the 3DP materials.

From Figure 8, it can readily be observed that the tensile strength is maximized for the PMMA/2% antibacterial nanocomposite, exceeding the value acquired for the PMMA pure by 20.2%. For the tensile modulus of elasticity, the maximum occurs for the PMMA/8% antibacterial nanocomposite, with a value that exceeds that of PMMA pure by 25.2%. On the contrary, the tensile toughness of all the nanocomposites is inferior when compared with PMMA pure.

The next step was to assess the flexural properties of the prepared nanocompounds. The mean and deviation values for the flexural strength, flexural modulus of elasticity, and toughness of all the 3DP materials are shown in Figure 9a–c. Almost the same trend as for the tensile values is also observed for the flexural values, namely the PMMA/4% antibacterial nanocomposite exhibits the most enhanced flexural strength among all the materials, showing a 10.8% increase for its value with respect to the PMMA pure. The PMMA/8% antibacterial nanocomposite shows the greatest value for the flexural modulus of elasticity, corresponding to a 25.1% increase when compared to the PMMA pure value. Lastly the values for the flexural toughness of the PMMA pure are the highest when compared to the other 3DP-prepared PMMA nanocomposites.

Finally, the flexural toughness of the various filaments was also investigated, along with the Charpy impact strength and the Vickers microhardness values, which are shown in Figure 10a–c, respectively. The filament toughness seems to have its maximum value for the PMMA pure, whereas for all PMMA—antibacterial nanocomposites the values seem to follow a systematic decrease. The Charpy impact strength seems to reach its highest value for the PMMA/8% antibacterial nanocomposite, an increase of 18.9% when compared to PMMA pure. Finally, the Vickers microhardness reaches a maximum value for the highest concentration PMMA—antibacterial nanocomposite, namely the PMMA/12% antibacterial nanocomposite, following a gradual increase starting from PMMA pure.

### 3.8. Examination of the Overall Morphology and Dimensional Characteristics of the PMMA—Antibacterial Nanocomposites Through μ-Computed High-Resolution Tomography

**Figure 11:**
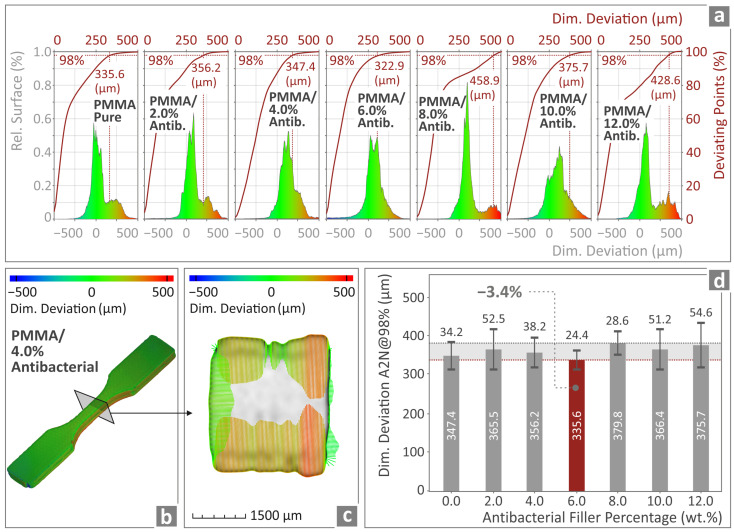
(**a**) Various graphs for comparing the differences between the computer-designed specimens and the actual 3DP ones for all PMMA-antibacterial nanocomposites. (**b**,**c**) Color-coded illustration of the tensile structural deviation of the PMMA/4% antibacterial nanocomposite. (**d**) A2N at the 98-dimensional deviation of the PMMA pure and various prepared PMMA—antibacterial nanocomposites.

Figure 11a presents various reluctant surface curves, along with the occurring deviating points vs. the dimensional deviation of the 3DP specimens when compared to the nominal ones from the design software, for all PMMA—antibacterial nanocomposites prepared and studied herein. Figure 11b,c show a color-coded illustration of the tensile structural deviation regarding the PMMA/4% antibacterial nanocomposite when actually 3DP and through the computer-assisted design (CAD) software. Remarkably, the deviation is almost negligible, ensuring this way that the material that has undergone 3DP is the same as the nominal designed ones. Figure 11d presents the deviation of the actual to nominal geometry, evaluating 98% of the measurements of PMMA pure and all subsequent PMMA—antibacterial nanocomposites. The best PMMA nanocomposite in this regard was the PMMA/6% antibacterial nanocomposite, which had 3.4% less deviation in the geometry when compared to PMMA pure, as shown in Figure 11d.

**Figure 12:**
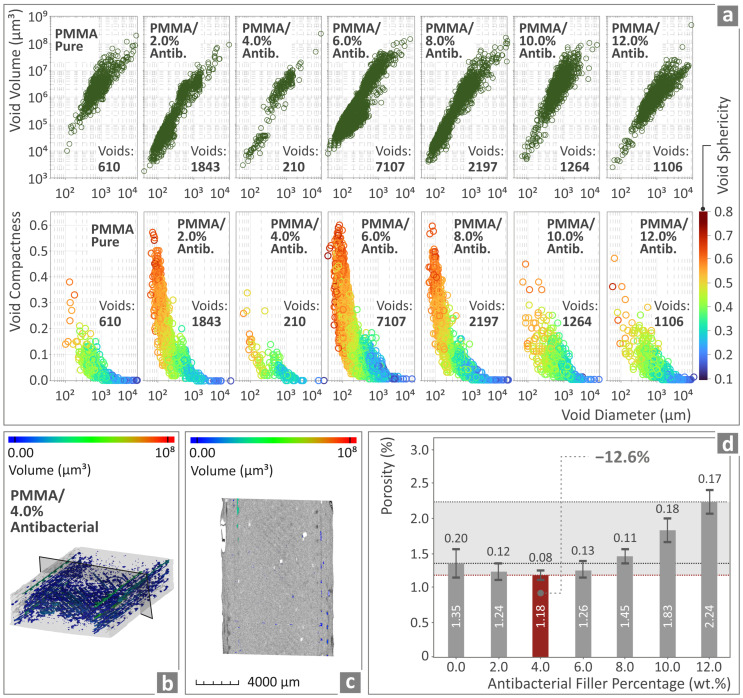
(**a**) Void’s compactness and sphericity vs. their diameter for all PMMA—antibacterial nanocomposites. (**b**,**c**) Color-coded illustration of the void distribution and volume for all PMMA—antibacterial nanocomposites that were 3DP. (**d**) Porosity levels of the PMMA pure and various prepared PMMA—antibacterial nanocomposites,.

The next step was to investigate the presence and formation of voids, the compactness, and the sphericity versus their diameter for the various prepared PMMA—antibacterial nanocomposites. These findings are presented in Figure 12a–d.

Figure 12a illustrates the voids’ compactness and sphericity vs. their diameter for all the different PMMA—antibacterial nanocomposites prepared via 3DP. Figure 12b,c show the relative void distributions for all the 3DP-prepared samples. Finally, the porosity percentage when compared to PMMA pure seems to reach its lowest value for the PMMA/4% antibacterial nanocomposite (12.6% less porosity for the nanocompound with 4 wt.% filler content). The porosity constantly increased as the filler content increased. It was found to be higher than in the pure PMMA parts at concentrations of 8 wt.% and higher.

### 3.9. SEM Investigation of the Morphological Characteristics of the PMMA—Antibacterial Nanocomposites

**Figure 13:**
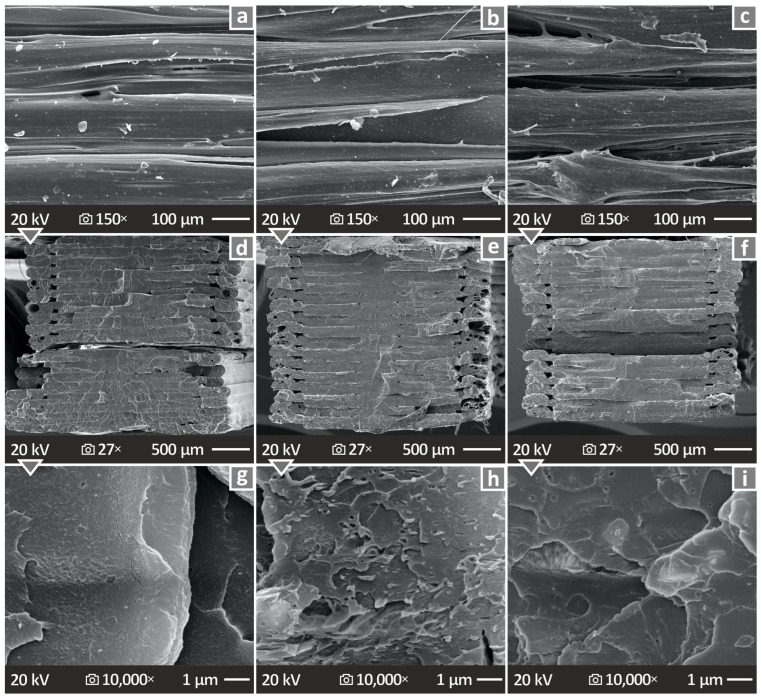
(**a**–**c**) PMMA/2%, 6%, and 10% at 150× magnification on their side. (**d**–**f**) PMMA/2%, 6%, and 10% at 27× magnification on their fracture side. (**g**–**i**) PMMA/2%, 6%, and 10% at 10,000× magnification on their fracture side.

**Figure 14:**
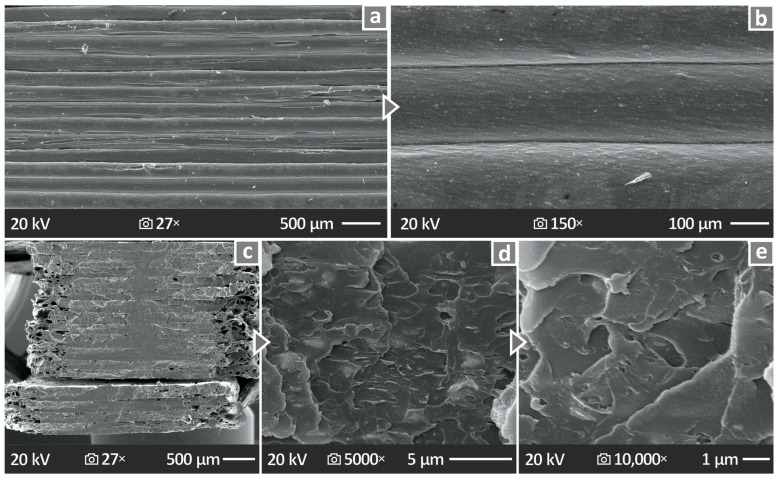
(**a**,**b**) PMMA/12% antibacterial nanocomposite at 27× magnification, acquired from the side. (**c**–**e**) PMMA/12% antibacterial nanocomposite at 27×, 500×, and 10,000× magnifications, respectively, acquired from the fracture side.

Various SEM illustrations acquired from the tensile samples after failure during mechanical tensile experiments from various specimens are shown in Figure 13 and Figure 14, respectively. Figure 13a–c show the SEM pictures captured from the PMMA/2%, 6%, and 10% antibacterial nanocomposites, respectively, at 150× magnification and viewing their side surface. On the same note, Figure 13d–f show the SEM images captured from fractured PMMA/2%, 6%, and 10% antibacterial nanocomposites, respectively, at 27× magnification and viewing their fractured surface. Finally, Figure 13g–i show the SEP images captured from fractured PMMA/2%, 6%, and 10% antibacterial nanocomposites at 10,000× magnification viewing their fractured surface. All the acquired images confirm that the 3DP materials with various loadings of the antibacterial nanopowder do not seem to exhibit any anomalous behavior and their overall surface, even when fractured, seems smooth and free of any impurities.

Figure 14a–e shows the SEM images acquired from the PMMA/12% antibacterial nanocomposite at various magnifications. Figure 14a,b show SEM images, at 27× and 150× magnifications, respectively, acquired from the side material. The next three SEM images for Figure 14, namely c–e, show the fracture surface at 27×, 5000×, and 10,000× magnifications, respectively. It is also observed through these SEM images that the material does not exhibit any peculiar behavior or any unwanted defects.

From the morphological analysis of the prepared PMMA—antibacterial nanocomposites, no major defects are observed. Even for the fracture surfaces, no residual cracking is observed and the separation surfaces do not seem to exhibit any significant deformation zones. This enhances the belief that the addition of the nanocomposites does not depreciate the quality of the 3DP materials. Even at the highest loading, no particle clustering was found in the SEM observations. In all cases, samples failed with a brittle failure, since no notable deformation was shown on the fracture section of the samples. In the images from the sides of the samples shown in Figure 13, the layer formation worsens with an increase in the filler percentage. Some gaps are shown on the 10 wt.% sample, which might have influenced the mechanical performance of the part.

### 3.10. Evaluating the Biocidal Capabilities of the PMMA—Antibacterial Nanocompounds

**Figure 15:**
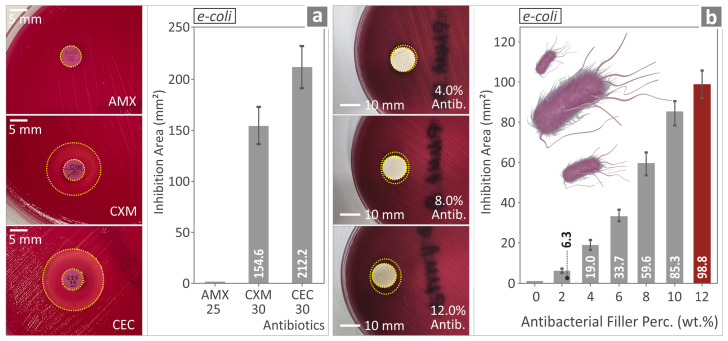
Antimicrobial efficacy outcome against *Ε. coli*, through IZ values, on (**a**) control samples, i.e., Amoxicillin, Cefuroxime, and Cefaclor, and (**b**) PMMA pure and antibacterial nanocomposites.

**Figure 16:**
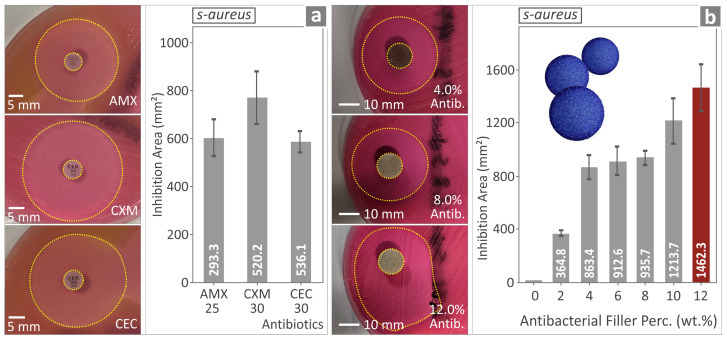
Antimicrobial efficacy outcome against *S. aureus*, through IZ values, on (**a**) control samples, i.e., Amoxicillin, Cefuroxime, and Cefaclor, and (**b**) PMMA pure and antibacterial nanocomposites.

The antibacterial performance of the various PMMA-antibacterial nanocomposites was evaluated, and their performance is shown in Figure 15 and Figure 16, a and b, respectively. The results shown in Figure 15a,b originate from the evaluation of the antibacterial properties of three (3) different antibiotic pills, namely Amoxicillin (AMX), Cefuroxime (CXM), and Cefaclor (CEC), respectively, against *Ε. coli*. Figure 16a,b evaluate the antibacterial properties of the same three (3) antibiotic pills against *S. aureus*.

*Ε. coli* showed no IZ for the AMX and the PMMA pure, while CXM and CEC showed larger IZs. For *S. aureus*, IZs were observed for all three antibiotics, namely AMX, CXM, and CEC, while the only nanocomposite with no IZ was again the PMMA pure. For *S. aureus,* the IZ was wide for the samples, much wider than in the case of *Ε. Coli*. The same trend was found in control antibiotics, depicting the consistency of the experimental outcome for the nanocomposites.

## 4. Discussion

In this work, we focused on the elucidation of the mechanical and antibacterial performance of several PMMA—antibacterial nanocomposites. The research was focused on mechanical, rheological, morphological, imaging, and biocidal testing to evaluate the multifunctionality of analogous 3DP materials.

The starting materials were PMMA pure, aiming to utilize it as a base model for all other investigations, and various other PMMA—antibacterial nanocomposites with different loadings of antibacterial nanopowder, namely 2%, 4%, 6%, 8%, 10%, and 12%, respectively. All these loadings are in wt.%, as mentioned above. To determine the optimal nanoparticle loading in the composites, the content was incrementally increased, with samples being synthesized and evaluated at each stage. The increase in loading was terminated when a decline in mechanical properties was observed, indicating that the filler had reached saturation within the nanocomposites [93,94]. Furthermore, higher loadings presented challenges for processing using the specific thermomechanical method employed for composite preparation. Issues with printability were encountered, resulting in suboptimal filament and sample quality. The gradual increase in the antibacterial nanopowder allows for a systematic evaluation of the biocidal properties of the corresponding PMMA—antibacterial nanocomposites, as any change in their behavior will be readily observed. We have focused our efforts on the range of 0–12 wt.% loadings, and we believe that any significant change will be observed in this range, as the loadings are gradually increasing, and not larger loadings.

A series of eighteen distinct tests were conducted to document and characterize the specific composites developed in the research. Over 190 samples were prepared and subjected to testing, with certain tests, such as rheology tests, not necessitating additional sample fabrication. Throughout the experimental process, the extrusion, 3D printing, and experimental conditions remained constant across all samples to ensure comparable results. The sole variable parameter was the filler content in the composites. Consequently, statistical analysis could not be performed in this instance. Future research may involve evaluating the significance of composite preparation parameters by testing them as control parameters at different levels.

Metallic nano-sized particles contribute to enhancing the load-bearing capacity of the polymer, thereby increasing the material’s stiffness and strength. The nanoparticles facilitate a more uniform and coherent stress distribution throughout the material, inhibiting localized deformation and improving overall performance. A crucial consideration is the interaction between metal nanoparticles and polymer chains. The nanoparticles establish an improved interfacial bond with the polymer matrix, which enhances stress transfer from the polymer chains to the nanoparticles. This interface has the potential to enhance the composite material’s strength by impeding crack propagation and increasing the material’s strength and toughness. The superior surface properties of metal nanoparticles can contribute to enhanced bonding and effective load transfer, thereby increasing the strength of the composite material. Overall, the incorporation of nanoparticles can lead to improved stress distribution and crack resistance due to reinforced interactions at the nanoparticle–polymer interface [109,110,111,112,113].

All the 3DP specimens show remarkable reproducibility and overall quality, as no significant deviations are observed with respect to the computer-designed structures. The best result is acquired for the PMMA/6% antibacterial nanocomposite. The porosity, on the other hand, seems to remain almost constant, reaching a minimum for the PMMA/4% antibacterial nanocomposite; however, for all higher-content antibacterial nanocomposites it seemed to steadily increase. This can possibly be explained by the possible segregation of the nanomaterials, creating in this way some areas where the porosity could increase. Porosity is expected in the 3D-printed structure. Even with 100% infill density, pores exist due to strand formation and overlap. So, this is common finding in 3D-printed parts. Porosity has direct effect on the mechanical properties, with higher values negatively affecting the mechanical performance [114,115]. How the porosity can impact the long-term durability and performance of the PMMA nanocomposites in real-world applications needs further investigation and can be the subject of a future work. The porosity can affect the long-term durability due to changes in the strand bonding over time. Still, the long-term durability of the 3D-printed parts is affected by several parameters other than the porosity, such as the mechanical properties, environmental resistance, and degradation behavior. The weather conditions and polymer aging highly affect the long-term durability and performance of the polymers, not just PMMA, and should also be considered in real-world applications [116].

The MFR seems to remain unaffected by the introduction of the antibacterial blend nanopowder into the PMMA polymer, which could explain why the various PMMA—antibacterial nanocomposites remain smooth and exhibit a high-quality filament production even when the antibacterial nanopowder is introduced into the PMMA pure. This is also confirmed by the very small variations observed for T_g_, as derived from the various DSC thermographs for all the prepared nanocomposites. The TGA results indicate no significant alterations in the thermal response of the PMMA due to the incorporation of the antibacterial blend nanoparticles. In the composite containing 10 wt.% filler, the temperature at which rapid degradation of the nanocomposites commences exhibited a slight increase, which, although seemingly minor, could potentially enhance thermal stability under prolonged exposure to high thermal stresses. This modest increase in the onset temperature of rapid degradation suggests an improvement in the thermal stability of the nanocomposites. Enhanced resistance to extended thermal stress may decelerate degradation, thereby contributing to the preservation of the material’s mechanical and structural integrity over time. Consequently, nanocomposites are becoming increasingly suitable for utilization in aerospace, automotive, and industrial applications where performance may be compromised due to thermal degradation. Furthermore, the improved tolerance to cyclic heating and cooling could potentially result in a reduction in thermal fatigue and facilitate greater long-term reliability in harsh environments. The residuals correspond to the filler content in the nanocompounds. Additionally, it was confirmed that the temperatures applied for the extrusion of the filament and the 3D printing of the samples were lower than the temperature at which rapid degradation of the nanocomposites initiates. Therefore, no degradation occurred in the nanocomposites that would adversely affect the mechanical properties of the 3D-printed nanocomposite components.

The introduction of the antibacterial nanocomposites seems to improve the overall mechanical strength of the PMMA—antibacterial nanocomposites (in agreement with other similar works in the literature [117]), except for toughness, which seems to be at the maximum for PMMA pure and gradually lowers for the other PMMA—antibacterial nanocomposites. The mechanical properties, such as the tensile strength, started to decrease beyond 2 wt.% loading. The nanocomposite with 8 wt.% loading showed comparable tensile strength to the unfilled PMMA thermoplastic, and the nanocomposites with higher loadings had tensile strengths lower than the pure PMMA. This is an indication that the filler has saturated the matrix, which leads to lower mechanical strength [93,94]. The research did not establish the saturation threshold. Since we wanted to improve the mechanical strength and induce antibacterial properties, there was no reason to further increase the filler content in the investigation.

The mechanical tests highlighted the nanocomposite with 2 wt.% content as the one with higher tensile strength among the various filler contents investigated. In the flexural tests, the 4 wt.% nanocompound was the one with the highest strength. Still, the value was very close to the one for the 2 wt.% nanocompound. Therefore, 2 wt.% can be considered the optimum loading regarding strength for the specific nanocompounds investigated herein. For the modulus of elasticity, the 8 wt.% nanocompound showed the most improved values in both the tensile and the flexural test. This shows that the PMMA becomes stiffer with the increase in the antibacterial blend nanoparticle content in the compounds but this does not lead to an increase in the strength as well. Still, such a response can be the looked-for specification in specific applications. Therefore, when the stiffness of the material is the required specification, higher filler loadings are suggested, and when the maximum strength is the main criterion, lower filler concentrations can achieve that. The decrease in strength at loadings higher than 2 wt.% shows that the particles failed to properly transfer the loads in the composites, although their distribution managed to make the composites stiffer.

It should be noted that, in the flexural tests, the samples failed before 5% strain was reached, where the respective standard instructs the termination of the experiment. This shows that the PMMA samples become even more brittle with the addition of the nanoparticles into the matrix. This agrees with the findings from the tensile tests as well. The PMMA pure was the one that failed at a higher strain, while the nanocompounds failed at lower strains. This explains the fact that the toughness was higher in the pure PMMA than in the nanocompounds, which means that the addition of the nanoparticles reduced the ability of the material to absorb energy during the test, hence the brittle failure of the samples. This is a common response when adding nanoparticles to polymeric matrices (brittleness increase and toughness reduction), as the literature reports [118,119,120,121]. On the other hand, the highest-loaded nanocompound was the one with the highest microhardness, which has merit in high-wear applications. This is due to the elements in the antibacterial blend nanoparticles, such as Zr, which have been reported to improve the hardness of polymeric materials [122].

The highest tensile and flexural toughness were found for the pure PMMA polymer. This shows that the addition of the nanoparticles, although increasing its strength, makes the polymer more brittle and with lower capability to absorb energy during testing. This should be considered when designing parts with these nanocomposites. Depending on the specific application, the optimal filler concentration of the PMMA/antibacterial nanocomposites can differ, especially if the biocidal properties should also be considered for the selection of the material.

Correlating the mechanical test results with the quality metrics assessed in the research, no clear connection can be derived between the dimensional accuracy and the mechanical strength. The nanocompound with 6 wt.% filler percentage was the one with the better geometrical fidelity, which did not reflect the mechanical performance. On the other hand, a clear correlation between the porosity and the mechanical response can be assumed. The 4 wt.% nanocompound was the one with the lowest porosity percentage and showed good mechanical performance among the nanocompounds investigated. The 2 wt.% nanocompound, which, overall, showed the highest mechanical response, was the one with the second lowest porosity, with a value very close to the 4 wt.% nanocompound. The highest-loaded nanocompound (12.0 wt.%) had the highest porosity, almost double the lowest value reported. It can be safely assumed that this reflected its mechanical properties, as its strength was lower than the pure PMMA (~15% in both the tensile and the flexural strength). It should be noted that, since the rheology of the PMMA polymer was not highly modified by the introduction of the antibacterial blend nanopowder, the increase in the porosity and the decrease in the mechanical performance cannot be attributed to changes in the rheology. If the rheology was affected, further adjustment in the 3D printing settings for the higher-loaded nanocompounds would be required to achieve a better-quality 3D printing structure. The rheological characteristics of the materials are important to achieve high-quality 3D printing parts with the MEX method [123,124].

Concerning the biocidal performance of the various PMMA—antibacterial nanocomposites manufactured, as expected, it is maximized for the nanocomposite with the highest concentration of antibacterial nanopowder. Overall, the introduction of the antibacterial nanoparticle blend into the PMMA polymeric matrix seems to even act beneficially on the mechanical properties of the PMMA nanocomposites, confirming that the production of reliable 3DP polymer nanocomposites is plausible and opens up novel opportunities for the medical field or the defense and security sector. These specific nanocomposites were found to be more efficient against *S. aureus* than *E. Coli*. This outcome agrees with the control examples assessed (Amoxicillin, Cefuroxime, and Cefaclor), depicting the consistency of the nanocompounds and the results provided by the screening method (agar well diffusion) employed for the evaluation of the biocidal performance of the developed nanocomposites in this research. The largest IZ was observed for the highest-loaded compound (12 wt.%).

Particle clustering can have a positive effect on the antibacterial performance, as the metallic parts’ concentration is locally increased and these parts are the ones responsible for the antibacterial properties in the nanocomposites. On the other hand, particle clustering negatively affects mechanical performance. Therefore, a balance between filler loading and filler clustering should be found to obtain nanocomposites with both good mechanical and biocidal performance. Still, the nanocomposite preparation method should be such that it ensures the good dispersion of the nanoparticles in the matrix. Herein, all the steps of process were toward the improvement of the filler dispersion in the matrix. For example, an extruder that was particularly useful for material mixing was used for the filament production, among other things. Furthermore, the dispersion of the nanoparticles in the matrix was checked through SEM and EDS mapping for the main elements of the antibacterial blend. Additionally, the deviation in the mechanical tests between the samples of the same batch was within acceptable limits, indicating a similar composition between the parts. SEM images did not reveal any particle clustering, even in the highest-loaded nanocomposite. This shows that a sufficient dispersion of the nanoparticles was achieved using the nanocomposite preparation method followed. Still, the SEM images verified that the assumption that the introduction of the nanoparticles makes the PMMA polymer more brittle, as the inspection of the fracture surfaces revealed.

Herein, no clear balance between antibacterial and mechanical performance can be derived, as the highest mechanical performance was achieved at lower filler loadings, as explained above. So, when the antibacterial properties are the priority, higher loadings should be selected, and when the mechanical performance is the key specification, low filler loadings should be preferred. Apart from the saturation phenomena of the additive at the highest nanocomposite, further increases in the filler loading led to inferior parts quality in the 3D printing process and processability issues, making the extrusion and the 3D printing of such nanocomposites more difficult.

The antibacterial capabilities of the specific nanopowder blend are due to its elements, such as Ag, which is known for its biocidal properties [125]. The mechanism to achieve this is not yet fully understood [99]. Still, the findings show that the ions of elements such as Ag penetrate the membrane of the bacteria cells, causing cell death [126]. The observed variation in the antibacterial performance of the PMMA nanocomposites against *E. coli* and *S. aureus* can be attributed to different factors. Metal nanoparticles have different antibacterial efficiencies. Positively charged nanocomposites interact more effectively with negatively charged bacterial cell membranes [127]. The difference in membrane composition between *E. coli* and *S. aureus* affects how nanocomposites bind and disrupt them [128]. Differences are expected; even in the control antibiotics differences were found in the effectiveness against the two pathogens and the nanocomposites’ response agrees with the response of the control samples (the same trend is reported for each bacterium), showing the reliability of the presented findings.

Still, due to the composition of the antibacterial blend, additional studies are needed as future work, for example in the cytotoxicity of the nanocomposites. Specific elements of the nanopowder blend, such as the phosphorus pentoxide (P_2_O_5_) [129,130] and silver (Ag) [125,131], are expected to cause cytotoxic effects. Therefore, such studies are necessary prior to the use of such nanocomposites in medical environments. Compliance with respective regulations should also be tested and verified.

The bibliography investigation, as mentioned above, revealed several studies on the antibacterial performance of PMMA-based composites; however, no similar research was found to directly correlate with the results of the current research. In the study by Rezaei et al. [68], the *E. Coli* bacterium was employed, as herein, to assess the antibacterial performance. Still, a different approach was used, so no direct comparison of the results is possible. Additionally, Rezaei’s research did not include mechanical tests. In the study by Bacali et al. [69], the same bacteria as used in the current research were tested using the same method and parameters. Graphene and silver particles were used as additives and the results agree with the current research regarding the biocidal performance. The biocidal performance was higher against *S. aureus* than *E. Coli*. Regarding the mechanical properties derived through flexural tests, an improvement was reported, similar to the current research, with the improvement percentage also being rather close. In the study by de Mori et al. [70], silver particles were also used and the biocidal behavior was tested against *S. aureus*. A different protocol (MTT assay) was applied, yet a significant reduction of the viable bacteria was found. Compression tests were carried out to evaluate the mechanical properties and an improvement was reported. In the study by Russo et al. [71], gold particles were evaluated against *S. aureus* and Pseudomonas Aeruginosa bacteria. The same method was used for bacterial incubation but the biocidal performance was evaluated with a different method, thus the results cannot be compared to the current investigation. Compression tests were performed for the evaluation of the mechanical properties. In the study by Marin et al. [73], nitrides were used as additives. The biocidal performance was evaluated with a different protocol, while no mechanical tests were performed. In the study by An et al. [74], Streptococcus mutans bacterium was evaluated. Zinc dimethacrylate was an additive and achieved an increase in flexural strength at 2.5 wt.%, with higher loading having reduced mechanical performance in the flexural experiment. The agar well diffusion method was used for the evaluation of the biocidal performance of the composites.

The literature review showed that different additives have been tested for their mechanical efficacy and antibacterial performance, as stated in the text. The studies showed that each additive achieved a different outcome in terms of mechanical and antibacterial performance. This is expected, since each additive has different capabilities and interacts with the polymeric matrix in a different way. This justifies the need for individual research works for each matrix/filler combination. In the presented works, composites were prepared with different methods and tested on different types of tests (mechanical and biocidal). Most of the research does not consider AM as well. In our case, this is an additional asset, as the method employed can be easily industrialized and refers to AM technology, which is very popular nowadays as a manufacturing method. Therefore, the results cannot be directly correlated to derive a solid conclusion regarding which additive is better overall in terms of mechanical or biocidal performance or both. Furthermore, specific additives are suitable and more common in specific types of applications. Depending on the application, the suitable matrix/filler combination can be selected as well as the preparation method for the composites (or nanocomposites depending on the size of the filler, which also lead to different effects). AM may or may not be a parameter in this equation. Therefore, each one of the existing studies should be evaluated depending on the specification of each application.

The possibility of having PMMA nanocomposites exhibiting biocidal properties that can be 3D printed enhances the potential for various uses in the defense and security sector, as many uses of PMMA are found in the medical field or emergency response situations. The 3D-printed parts can significantly lower the cost of manufacturing but also provide the ability for on-site production of various materials, minimizing in this way the need for large storage units or time-consuming logistics lines. The latter, especially in an operational environment, is of crucial importance, as it can expose significant vulnerabilities in the military supply chain.

Another very important parameter for having the ability to 3DP various PMMA—antibacterial nanocomposites arises in the domain of producing personalized parts that can maximize usage and operational effectiveness when compared to the “one size, fits all” approach. This is vividly evident in medical implants, where several anatomical variations might arise. Having the ability to 3DP personalized equipment with antibacterial multifunctionality can certainly minimize the treatment time, possibility of infections, and/or risk of fatalities due to severe injuries and mistreatment of wounds.

PMMA is a widely used thermoplastic polymer with known biocompatibility and several uses in the medical domain, as stated before. The novel approach presented here for enhancing both the mechanical properties and inducing antibacterial properties can open up several new opportunities for the defense industry on many different levels. This ranges from medical equipment for medical emergencies on the battlefield to providing opportunities for the arms industry regarding the production of the various parts that are placed on handheld firearms, thus minimizing the possibility of infections when the weapon is used by several individuals. In this direction, our findings presented herein provide a solid foundation for further investigation on the possibilities of introducing antibacterial nanocomposites to polymeric matrices aiming to manufacture various materials that can have profound uses in the defense and security domain but also have a “dual use” character in the medical and emergency response field and civil protection. The limitations are mainly related to the investigation of the antibacterial properties, in which more advanced methods following respective standards, such as ISO 22196, should also be applied to obtain more solid results on this issue. Additionally, the cytotoxicity of the prepared nanocomposites was not addressed. Future work can address these issues.

## 5. Conclusions

In this work, we have successfully synthesized a series of PMMA—antibacterial nanocomposites as high-quality filaments and successfully manufactured several 3DP specimens exhibiting simultaneously enhanced mechanical properties and biocidal activity when compared to PMMA pure 3DP materials. In this regard, we were able to systematically follow the influence of incorporating various loadings of antibacterial nanopowder into the PMMA polymer matrix. The pathogens that were used in order to confirm biocidal activity were *E. coli* and *S. aureus*, two of the most common pathogens deteriorating human health. Our findings confirmed the multifunctionality (enhanced mechanical response and biocidal performance) of the 3DP PMMA nanocomposites and opened up the road for more intensive investigation as to how PMMA might behave when introduced into its polymer matrix and other antibacterial nanocomposites, depending on the desired multifunctionality. The 2 wt.% filler content can be considered the optimum loading regarding the mechanical strength. When biocidal performance is the main requirement, the maximum loading of 12 wt.% achieved the best results. It should be noted that the performance against *E. coli* and *S. aureus* differed, with the nanocomposites being more effective against the latter, which was in agreement with the control samples evaluated.

In this respect, it is within the scope of our work to further investigate the possibility of controlled drug release from 3DP samples, as they can provide very useful multi-functionalities in cases where severe injuries occur along with burn injuries, such as the ones commonly found in multi-traumatized personnel operating in urban hot military zones where acute combat occurs. In such cases, it is important to treat several fractures that have originated from blasting debris and at the same time treat severe burns caused by the blast. The first injury category needs immobilization of the broken part of the body but the second injury needs continuous care of the burned skin and tissue through the application of hydration gels and inflammation reducers. These two needs can be excellently combined when 3DP materials can also exhibit controlled release of medication. Our work will continue in this direction, as it will contribute towards saving lives on the battlefield and minimizing the effects of severe injuries originating from any type of event. In future work, additional bacteria can be tested and more focused studies can be carried out for the biocidal performance of the nanocomposites, while cytotoxicity studies can provide useful information as well. Aspects such as compliance with medical regulations and standards can be also evaluated. Furthermore, the industrialization of the process can be studied to locate and address the challenges involved. Additionally, long-term durability testing can be implemented to assess the performance of the presented nanocomposites over time. Other aspects can be assessed as well, such as the impact of nanoparticle dispersion on the final properties of the nanocomposites, including the mechanical, thermal, and biocidal properties. This could also be a crucial area for further investigation.

## Figures and Tables

**Table 1:** Significant Raman peak differences between PMMA pure versus PMMA/antibacterial 2 wt.%, 4 wt.%, 6 wt.%, 8 wt.%, 10 wt.%, and 12 wt.%.

603	Increase	Small increase in all PMMA/antibacterial samples
814	Gradual Increase	Medium gradual increase
1088	Gradual Increase	Medium gradual increase
1286	Increase	Small increase
1614	Gradual Increase	Medium gradual increase with the exception of a large increase in the 6% sample Please check beam
1727	Gradual Increase	Medium gradual increase with the exception of a large increase in the 6%sample
2925	Decrease	Medium decrease appearing in all samples

## Data Availability

The raw/processed data required to reproduce these findings cannot be shared because of technical and temporal limitations.

## Supplementary Materials

The following supporting information can be downloaded at: https://www.mdpi.com/article/10.3390/polym17030410/s1, Figure S1. For all the PMMA/antibacterial blend compounds tested: (a) Tensile stress vs. strain curve. (b) Flexural stress vs. strain curve. (c) Filament tensile stress vs. strain curve.; Figure S2. Settings used for parts manufacturing with MEX 3D printing and the geometry of the samples manufactured, following the respective standards. The infill pattern of the 3D printing structure is also indicated.; Figure S3. Spider graph summarizing the main experimental findings. The highest values are indicated.; Table S1. Significant Raman peaks and their related assignments from PMMA pure.

## References

[1] Dittenber D.B., GangaRao H.V.S. (2012). Critical Review of Recent Publications on Use of Natural Composites in Infrastructure. Compos. Part A Appl. Sci. Manuf..

[2] Nielsen T.D., Cruickshank C., Foged S., Thorsen J., Krebs F.C. (2010). Business, Market and Intellectual Property Analysis of Polymer Solar Cells. Sol. Energy Mater. Sol. Cells.

[3] Cheah C.M., Chua C.K., Lee C.W., Feng C., Totong K. (2005). Rapid Prototyping and Tooling Techniques: A Review of Applications for Rapid Investment Casting. Int. J. Adv. Manuf. Technol..

[4] Ngo T.D., Kashani A., Imbalzano G., Nguyen K.T.Q., Hui D. (2018). Additive Manufacturing (3D Printing): A Review of Materials, Methods, Applications and Challenges. Compos. Part B Eng..

[5] Frazier W.E. (2014). Metal Additive Manufacturing: A Review. J. Mater. Eng. Perform..

[6] Yap C.Y., Chua C.K., Dong Z.L., Liu Z.H., Zhang D.Q., Loh L.E., Sing S.L. (2015). Review of Selective Laser Melting: Materials and Applications. Appl. Phys. Rev..

[7] Wang X., Jiang M., Zhou Z., Gou J., Hui D. (2017). 3D Printing of Polymer Matrix Composites: A Review and Prospective. Compos. Part B Eng..

[8] Dizon J.R.C., Espera A.H., Chen Q., Advincula R.C. (2018). Mechanical Characterization of 3D-Printed Polymers. Addit. Manuf..

[9] Parandoush P., Lin D. (2017). A Review on Additive Manufacturing of Polymer-Fiber Composites. Compos. Struct..

[10] Bagheri A., Jin J. (2019). Photopolymerization in 3D Printing. ACS Appl. Polym. Mater..

[11] Lambert-Perlade A., Gourgues A.F., Besson J., Sturel T., Pineau A. (2004). Mechanisms and Modeling of Cleavage Fracture in Simulated Heat-Affected Zone Microstructures of a High-Strength Low Alloy Steel. Metall. Mater. Trans. A.

[12] Zhang L.-J., Pei Q., Zhang J.-X., Bi Z.-Y., Li P.-C. (2014). Study on the Microstructure and Mechanical Properties of Explosive Welded 2205/X65 Bimetallic Sheet. Mater. Des..

[13] Angrish A. A Critical Analysis of Additive Manufacturing Technologies for Aerospace Applications. Proceedings of the 2014 IEEE Aerospace Conference.

[14] Weyhrich C.W., Long T.E. (2022). Additive Manufacturing of High-Performance Engineering Polymers: Present and Future. Polym. Int..

[15] Bird D.T., Ravindra N.M. (2021). Additive Manufacturing of Sensors for Military Monitoring Applications. Polymers.

[16] Michailidis N., Petousis M., Moutsopoulou A., Argyros A., Ntintakis I., Papadakis V., Nasikas N.K., Vidakis N. (2024). Engineering Response of Biomedical Grade Isotactic Polypropylene Reinforced with Titanium Nitride Nanoparticles for Material Extrusion Three-Dimensional Printing. Eur. J. Mater..

[17] Nasikas N.K., Petousis M., Papadakis V., Argyros A., Valsamos J., Gkagkanatsiou K., Sagris D., David C., Michailidis N., Maravelakis E. (2024). A Comprehensive Optimization Course of Antimony Tin Oxide Nanofiller Loading in Polyamide 12: Printability, Quality Assessment, and Engineering Response in Additive Manufacturing. Nanomaterials.

[18] Petousis M., Michailidis N., Saltas V., Papadakis V., Spiridaki M., Mountakis N., Argyros A., Valsamos J., Nasikas N.K., Vidakis N. (2024). Mechanical and Electrical Properties of Polyethylene Terephthalate Glycol/Antimony Tin Oxide Nanocomposites in Material Extrusion 3D Printing. Nanomaterials.

[19] Vidakis N., Petousis M., Michailidis N., Papadakis V., Mountakis N., Argyros A., Dimitriou E., Charou C., Moutsopoulou A. (2023). Polylactic Acid/Silicon Nitride Biodegradable and Biomedical Nanocomposites with Optimized Rheological and Thermomechanical Response for Material Extrusion Additive Manufacturing. Biomed. Eng. Adv..

[20] Vidakis N., Petousis M., Michailidis N., Nasikas N., Papadakis V., Argyros A., Mountakis N., Charou C., Moutsopoulou A. (2023). Optimizing Titanium Carbide (TiC) Ceramic Nanofiller Loading in Isotactic Polypropylene for MEX Additive Manufacturing: Mechano-Thermal and Rheology Aspects. Mater. Today Commun..

[21] Michailidis N., Petousis M., Saltas V., Papadakis V., Spiridaki M., Mountakis N., Argyros A., Valsamos J., Nasikas N.K., Vidakis N. (2024). Investigation of the Effectiveness of Silicon Nitride as a Reinforcement Agent for Polyethylene Terephthalate Glycol in Material Extrusion 3D Printing. Polymers.

[22] Petousis M., Michailidis N., Papadakis V., Argyros A., Spiridaki M., Mountakis N., Valsamos J., Nasikas N.K., Moutsopoulou A., Vidakis N. (2024). Enhanced Engineering and Biocidal Polypropylene Filaments Enabling Melt Reduction of AgNO_3_ through PVP Agent: A Scalable Process for the Defense Industry with MEX Additive Manufacturing. Def. Technol..

[23] Vidakis N., Petousis M., David C., Nasikas N.K., Sagris D., Mountakis N., Spiridaki M., Moutsopoulou A., Stratakis E. (2024). Critical Quality Indicators of High-Performance Polyetherimide (ULTEM) over the MEX 3D Printing Key Generic Control Parameters: Prospects for Personalized Equipment in the Defense Industry. Def. Technol..

[24] Vidakis N., Michailidis N., Petousis M., Nasikas N.K., Saltas V., Papadakis V., Mountakis N., Argyros A., Spiridaki M., Valsamos I. (2024). Multifunctional HDPE/Cu Biocidal Nanocomposites for MEX Additive Manufactured Parts: Perspectives for the Defense Industry. Def. Technol..

[25] Xue J., Hou Y., Chu W., Zhang Z., Dong Z., Zhang L., Wen G. (2024). 3D Printed B4C-Based Honeycomb Ceramic Composite and Its Potential Application in Three-Dimensional Armor Structure. Chem. Eng. J..

[26] Du Z., Chen C., Wang X. (2024). Study on the Mechanism and Performance of 3D-Printed PLA/Epoxy Composite for Stab Resistance. Rapid Prototyp. J..

[27] Sitotaw D.B., Muenks D., Kebash A.K. (2024). 3D Printing Applications on Textiles: Measurement of Air Permeability for Potential Use in Stab-Proof Vests. J. Eng. Fiber Fabr..

[28] Vidakis N., Petousis M., Michailidis N., David C., Saltas V., Sagris D., Spiridaki M., Argyros A., Mountakis N., Papadakis V. (2024). Interpretation of the Optimization Course of Silicon Nitride Nano-Powder Content in Biomedical Resins for Vat Photopolymerization Additive Manufacturing. Ceram. Int..

[29] Aktitiz İ., Aydın K., Darıcık F., Topcu A. (2022). Production of Different Metal Oxide Nanoparticle Embedded Polymer Matrix Composite Structures by the Additive Manufacturing Technology and Investigation of Their Properties. Polym. Compos..

[30] Cano S., Gooneie A., Kukla C., Rieß G., Holzer C., Gonzalez-Gutierrez J. (2020). Modification of Interfacial Interactions in Ceramic-Polymer Nanocomposites by Grafting: Morphology and Properties for Powder Injection Molding and Additive Manufacturing. Appl. Sci..

[31] Billings C., Cai C., Liu Y. (2021). Utilization of Antibacterial Nanoparticles in Photocurable Additive Manufacturing of Advanced Composites for Improved Public Health. Polymers.

[32] Vidakis N., Petousis M., David C.N., Sagris D., Mountakis N. (2023). Biomedical Resin Reinforced with Cellulose Nanofibers (CNF) in VAT Photopolymerization (VPP) Additive Manufacturing (AM): The Effect of Filler Loading and Process Control Parameters on Critical Quality Indicators (CQIs). J. Manuf. Process..

[33] Kwon H., Park S., Kwon S., Lee H.-T. (2023). Effect of the Cellulose Nanofiber on Ultraviolet Curable Resin for Additive Manufacturing: Mechanical Properties and Printability. Addit. Manuf..

[34] Mohan D., Teong Z.K., Bakir A.N., Sajab M.S., Kaco H. (2020). Extending Cellulose-Based Polymers Application in Additive Manufacturing Technology: A Review of Recent Approaches. Polymers.

[35] Gauss C., Pickering K.L. (2023). A New Method for Producing Polylactic Acid Biocomposites for 3D Printing with Improved Tensile and Thermo-Mechanical Performance Using Grafted Nanofibrillated Cellulose. Addit. Manuf..

[36] Dong J., Mei C., Han J., Lee S., Wu Q. (2019). 3D Printed Poly(Lactic Acid) Composites with Grafted Cellulose Nanofibers: Effect of Nanofiber and Post-Fabrication Annealing Treatment on Composite Flexural Properties. Addit. Manuf..

[37] Yao K., Hong G., Yuan X., Kong W., Xia P., Li Y., Chen Y., Liu N., He J., Shi J. (2024). 3D Printing of Tough Hydrogel Scaffolds with Functional Surface Structures for Tissue Regeneration. Nano-Micro Lett..

[38] Zhu S., Zhou Z., Chen X., Zhu W., Yang M., Yu M., Sun J., Zuo Y., He J., Pan H. (2025). High Mechanical Performance and Multifunctional Degraded Fucoidan-Derived Bioink for 3D Bioprinting. Carbohydr. Polym..

[39] Trivedi A.K., Gupta M.K. (2024). PLA Based Biodegradable Bionanocomposite Filaments Reinforced with Nanocellulose: Development and Analysis of Properties. Sci. Rep..

[40] Mirzavandi Z., Poursamar S.A., Amiri F., Bigham A., Rafienia M. (2024). 3D Printed Polycaprolactone/Gelatin/Ordered Mesoporous Calcium Magnesium Silicate Nanocomposite Scaffold for Bone Tissue Regeneration. J. Mater. Sci. Mater. Med..

[41] Kaul N., Cachelin S. (2024). Non-Lethal Weapons and the Sensory Repression of Dissent in Democracies. Secur. Dialogue.

[42] Robbe C., Papy A., Nsiampa N., Drapela P., Bir C. (2023). NATO Standardized Method for Assessing the Thoracic Impact of Kinetic Energy Non-Lethal Weapons. Hum. Factors Mech. Eng. Def. Saf..

[43] Guo Z., Poot A.A., Grijpma D.W. (2021). Advanced Polymer-Based Composites and Structures for Biomedical Applications. Eur. Polym. J..

[44] Fatima N., Qazi U.Y., Mansha A., Bhatti I.A., Javaid R., Abbas Q., Nadeem N., Rehan Z.A., Noreen S., Zahid M. (2021). Recent Developments for Antimicrobial Applications of Graphene-Based Polymeric Composites: A Review. J. Ind. Eng. Chem..

[45] Luo H., Yin X.-Q., Tan P.-F., Gu Z.-P., Liu Z.-M., Tan L. (2021). Polymeric Antibacterial Materials: Design, Platforms and Applications. J. Mater. Chem. B.

[46] Maruthapandi M., Saravanan A., Gupta A., Luong J.H.T., Gedanken A. (2022). Antimicrobial Activities of Conducting Polymers and Their Composites. Macromol.

[47] Avcu E., Bastan F.E., Guney M., Yildiran Avcu Y., Ur Rehman M.A., Boccaccini A.R. (2022). Biodegradable Polymer Matrix Composites Containing Graphene-Related Materials for Antibacterial Applications: A Critical Review. Acta Biomater..

[48] Satanovsky A., Gilor Y., Benov A., Chen J., Shlaifer A., Talmy T., Radomislensky I., Siman-Tov M., Peleg K., Weil Y.A. (2024). Combat Injury Profile in Urban Warfare. Mil. Med..

[49] Sanyal A., Roy S., Ghosh A., Chakraborty M., Ghosh A., Mandal D. (2024). The next Frontier in Hemorrhagic Management: A Comprehensive Review on Development of Natural Polymer-Based Injectable Hydrogels as Promising Hemostatic Dressings. Chem. Eng. J..

[50] Pasquier P., David M., Petit L., Chery M., Habas S., Patey E., Conort S., Zeller N., Gelmann M.-O., Peyrefitte S. (2024). Irregular Warfare Must Combine Good Medicine, with Both Good Tactics and Good Strategies: Position Paper by the French Special Operations Forces Medical Command. J. Trauma Acute Care Surg..

[51] Singh S., Ramakrishna S., Singh R. (2017). Material Issues in Additive Manufacturing: A Review. J. Manuf. Process..

[52] Bikas H., Stavropoulos P., Chryssolouris G. (2016). Additive Manufacturing Methods and Modelling Approaches: A Critical Review. Int. J. Adv. Manuf. Technol..

[53] Yadav S., Liu S., Singh R.K., Sharma A.K., Rawat P. (2024). A State-of-Art Review on Functionally Graded Materials (FGMs) Manufactured by 3D Printing Techniques: Advantages, Existing Challenges, and Future Scope. J. Manuf. Process..

[54] Boretti A. (2024). A Techno-Economic Perspective on 3D Printing for Aerospace Propulsion. J. Manuf. Process..

[55] Atalie D., Guo Z.-S., Berihun D., Tadesse M., Ma P.-C., Rangappa S.M., Ayyappan V., Siengchin S. (2024). 20—Role of Additive Manufacturing in Defense Technologies: Emerging Trends and Future Scope. Additive Manufacturing Materials and Technology.

[56] Chaloupka K., Malam Y., Seifalian A.M. (2010). Nanosilver as a New Generation of Nanoproduct in Biomedical Applications. Trends Biotechnol..

[57] Klaus T., Joerger R., Olsson E., Granqvist C.-G. (1999). Silver-Based Crystalline Nanoparticles, Microbially Fabricated. Proc. Natl. Acad. Sci. USA.

[58] Bellisario D., Santo L., Quadrini F., Hassiba M., Bader N., Chowdhury S.H., Hassan M.K., Zughaier S.M. (2023). Cytotoxicity and Antibiofilm Activity of Silver-Polypropylene Nanocomposites. Antibiotics.

[59] Kumar C.S.S.R. (2010). Nanocomposites.

[60] Salam O.A., Hamad H.A., Eltokhy M.A.R., Ali A.I., Son J.Y., Ramzy G.H. (2024). A Comparative Study of PMMA/PEG Polymer Nanocomposites Doped with Different Oxides Nanoparticles for Potential Optoelectronic Applications. Sci. Rep..

[61] Kim S.-I., Moon J.-Y., Hyeong S.-K., Ghods S., Kim J.-S., Choi J.-H., Park D.S., Bae S., Cho S.H., Lee S.-K. (2024). Float-Stacked Graphene–PMMA Laminate. Nat. Commun..

[62] Ahn C.H., Zhang G., Suo Z. (2024). Ductility of a Nanocomposite of Glassy and Rubbery Polymers. J. Mech. Phys. Solids.

[63] Zong Y., Wu K., Fang W., Yu J., Jiang C., Xu G. (2023). Modified Intrascleral Fixation for Repositioning the Dislocated Single-Piece, Rigid PMMA Intraocular Lens. RETINA.

[64] Ahmed M.A.M., Jurczak K.M., Lynn N.S., Mulder J.-P.S.H., Verpoorte E.M.J., Nagelkerke A. (2024). Rapid Prototyping of PMMA-Based Microfluidic Spheroid-on-a-Chip Models Using Micromilling and Vapour-Assisted Thermal Bonding. Sci. Rep..

[65] Obaeed N.H., Hamdan W.K. (2024). Reconstruction and Evaluation of 3D Printing PMMA Cranioplasty Implants. Int. J. Interact. Des. Manuf. (IJIDeM).

[66] Durkin A.J., Catena D., Woltjen N., Boyle K., Polling M., Weng J., Chim J.H. (2023). Surgical Management of Polymethylmethacrylate-Collagen Gel Complications in the Lower Eyelid: A Case Series. Ann. Plast. Surg..

[67] Kumari S., Mishra R.K., Parveen S., Avinashi S.K., Hussain A., Kumar S., Banerjee M., Rao J., Kumar R., Gautam R.K. (2024). Fabrication, Structural, and Enhanced Mechanical Behavior of MgO Substituted PMMA Composites for Dental Applications. Sci. Rep..

[68] Rezaei F., Abbasi-Firouzjah M., Shokri B. (2014). Investigation of Antibacterial and Wettability Behaviours of Plasma-Modified PMMA Films for Application in Ophthalmology. J. Phys. D Appl. Phys..

[69] Bacali C., Baldea I., Moldovan M., Carpa R., Olteanu D.E., Filip G.A., Nastase V., Lascu L., Badea M., Constantiniuc M. (2020). Flexural Strength, Biocompatibility, and Antimicrobial Activity of a Polymethyl Methacrylate Denture Resin Enhanced with Graphene and Silver Nanoparticles. Clin. Oral Investig..

[70] De Mori A., Di Gregorio E., Kao A.P., Tozzi G., Barbu E., Sanghani-Kerai A., Draheim R.R., Roldo M. (2019). Antibacterial PMMA Composite Cements with Tunable Thermal and Mechanical Properties. ACS Omega.

[71] Russo T., Gloria A., De Santis R., D’Amora U., Balato G., Vollaro A., Oliviero O., Improta G., Triassi M., Ambrosio L. (2017). Preliminary Focus on the Mechanical and Antibacterial Activity of a PMMA-Based Bone Cement Loaded with Gold Nanoparticles. Bioact. Mater..

[72] Su W., Wang S., Wang X., Fu X., Weng J. (2010). Plasma Pre-Treatment and TiO_2_ Coating of PMMA for the Improvement of Antibacterial Properties. Surf. Coat. Technol..

[73] Marin E., Boschetto F., Zanocco M., Honma T., Zhu W., Pezzotti G. (2021). Explorative Study on the Antibacterial Effects of 3D-Printed PMMA/Nitrides Composites. Mater. Des..

[74] An J., Ding N., Zhang Z. (2022). Mechanical and Antibacterial Properties of Polymethyl Methacrylate Modified with Zinc Dimethacrylate. J. Prosthet. Dent..

[75] Vidakis N., Petousis M., Mountakis N., Moutsopoulou A., Karapidakis E. (2023). Energy Consumption vs. Tensile Strength of Poly [Methyl Methacrylate] in Material Extrusion 3D Printing: The Impact of Six Control Settings. Polymers.

[76] Song S., Lin X., Ming X., Lu D., Sun M., Wang B., Bao C., Lu B., Ma E. (2025). PMMA Light Channels Facilitate the Additive Manufacturing of Complex-Structured SiC Reflector Mirrors. J. Eur. Ceram. Soc..

[77] Mikhalchan A., Tay T.E., Banas A.M., Banas K., Breese M.B.H., Borkowska A.M., Nowakowski M., Kwiatek W.M., Paluszkiewicz C. (2020). Development of Continuous CNT Fibre-Reinforced PMMA Filaments for Additive Manufacturing: A Case Study by AFM-IR Nanoscale Imaging. Mater. Lett..

[78] Hata K., Ikeda H., Nagamatsu Y., Masaki C., Hosokawa R., Shimizu H. (2021). Development of Dental Poly(Methyl Methacrylate)-Based Resin for Stereolithography Additive Manufacturing. Polymers.

[79] Zafar M.S. (2020). Prosthodontic Applications of Polymethyl Methacrylate (PMMA): An Update. Polymers.

[80] Petersmann S., Hentschel L., Gonzalez-Gutierrez J., Tödtling M., Schäfer U., Arbeiter F., Üçal M. (2023). The Effects of Washing and Formaldehyde Sterilization on the Mechanical Performance of Poly(Methyl Methacrylate) (PMMA) Parts Produced by Material Extrusion-Based Additive Manufacturing or Material Jetting. Adv. Eng. Mater..

[81] Puppi D., Morelli A., Bello F., Valentini S., Chiellini F. (2018). Additive Manufacturing of Poly(Methyl Methacrylate) Biomedical Implants with Dual-Scale Porosity. Macromol. Mater. Eng..

[82] Stenzler J.S., Goulbourne N.C. (2011). The Effect of Polyacrylate Microstructure on the Impact Response of PMMA/PC Multi-Laminates. Int. J. Impact Eng..

[83] Xia F., Yang L., Dai B., Gao G., Yang Z., Cao W., Xu L., Geng F., Song Z., Ralchenko V. (2020). Novel Reparation Method for Polymethyl Methacrylate Optical Windows of Aircrafts Damaged by Service Environment. Sci. China Technol. Sci..

[84] Jia H., Li X., Fan Y., Ding C., Pan L., Feng X., Liu X., Hu J., Chen J., Gao L. (2020). Highly Efficient Broadband Solar-Blind UV Photodetector Based on Gd2O3:Eu3+–PMMA Composite Film. Adv. Mater. Interfaces.

[85] Tang E., Wang D., Li L., Peng H., Han Y., Chen C., Chang M., Guo K., He L. (2023). Polarization Response Characteristics of 6061Al and PMMA Sheets under Impact Load. Int. J. Impact Eng..

[86] Torres S.M., Vorobiev O.Y., Robey R.E., Hargather M.J. (2023). A Study of Explosive-Induced Fracture in Polymethyl Methacrylate (PMMA). J. Appl. Phys..

[87] Torres S.M., Hargather M.J., Kimberley J., Robey R.E. (2024). Shock Response of Polymethyl Methacrylate (PMMA) Under Explosive Loading. J. Dyn. Behav. Mater..

[88] Kazarinov N.A., Bratov V.A., Morozov N.F., Petrov Y.V., Balandin V.V., Iqbal M.A., Gupta N.K. (2020). Experimental and Numerical Analysis of PMMA Impact Fracture. Int. J. Impact Eng..

[89] Wang X., Xu J., Chen S., Li Y., Gao G., Gu W., Sha M. (2023). Damage Behavior and Assessment of Aeronautical PMMA Subjected to High-Velocity Water-Jet Impact. Wear.

[90] Sadeghi Esfahlani S. (2021). Ballistic Performance of Polycarbonate and Polymethyl Methacrylate under Normal and Inclined Dynamic Impacts. Heliyon.

[91] Seo J.Y., Choi M.H., Lee B.W., Lee J.-H., Shin S., Cho S., Cho K.Y., Baek K.-Y. (2022). Feasible Detoxification Coating Material for Chemical Warfare Agents Using Poly(Methyl Methacrylate)–Branched Poly(Ethyleneimine) Copolymer and Metal–Organic Framework Composites. ACS Appl. Mater. Interfaces.

[92] Yousfi M., Belhadj A., Lamnawar K., Maazouz A. 3D Printing of PLA and PMMA Multilayered Model Polymers: An Innovative Approach for a Better-Controlled Pellet Multi-Extrusion Process. Proceedings of the ESAFORM 2021, 24th International Conference on Material Forming.

[93] Song Y., Zheng Q. (2016). Concepts and Conflicts in Nanoparticles Reinforcement to Polymers beyond Hydrodynamics. Prog. Mater. Sci..

[94] Zare Y., Rhee K.Y., Hui D. (2017). Influences of Nanoparticles Aggregation/Agglomeration on the Interfacial/Interphase and Tensile Properties of Nanocomposites. Compos. Part B Eng..

[95] Balouiri M., Sadiki M., Ibnsouda S.K. (2016). Methods for in Vitro Evaluating Antimicrobial Activity: A Review. J. Pharm. Anal..

[96] Sedlarik V., Galya T., Sedlarikova J., Valasek P., Saha P. (2010). The Effect of Preparation Temperature on the Mechanical and Antibacterial Properties of Poly(Vinyl Alcohol)/Silver Nitrate Films. Polym. Degrad. Stab..

[97] Vidakis N., Petousis M., Velidakis E., Mountakis N., Grammatikos S.A., Tzounis L. (2023). Multi-Functional Medical Grade Polyamide12/Carbon Black Nanocomposites in Material Extrusion 3D Printing. Compos. Struct..

[98] Gomaa E.Z. (2017). Silver Nanoparticles as an Antimicrobial Agent: A Case Study on *Staphylococcus aureus* and *Escherichia coli* as Models for Gram-Positive and Gram-Negative Bacteria. J. Gen. Appl. Microbiol..

[99] Feng Q.L., Wu J., Chen G.Q., Cui F.Z., Kim T.N., Kim J.O. (2000). A Mechanistic Study of the Antibacterial Effect of Silver Ions on *Escherichia coli* and *Staphylococcus aureus*. J. Biomed. Mater. Res..

[100] Leber A.L. (2020). Clinical Microbiology Procedures Handbook.

[101] Veluthandath A.V., Bisht P.B. (2017). Identification of Whispering Gallery Mode (WGM) Coupled Photoluminescence and Raman Modes in Complex Spectra of MoS2 in Polymethyl Methacrylate (PMMA) Microspheres. J. Lumin..

[102] Zimmerer C., Matulaitiene I., Niaura G., Reuter U., Janke A., Boldt R., Sablinskas V., Steiner G. (2019). Nondestructive Characterization of the Polycarbonate—Octadecylamine Interface by Surface Enhanced Raman Spectroscopy. Polym. Test..

[103] Stuart B.H. (1996). Temperature Studies of Polycarbonate Using Fourier Transform Raman Spectroscopy. Polym. Bull..

[104] Resta V., Quarta G., Lomascolo M., Maruccio L., Calcagnile L. (2015). Raman and Photoluminescence Spectroscopy of Polycarbonate Matrices Irradiated with Different Energy 28Si+ Ions. Vacuum.

[105] Makarem M., Lee C.M., Kafle K., Huang S., Chae I., Yang H., Kubicki J.D., Kim S.H. (2019). Probing Cellulose Structures with Vibrational Spectroscopy. Cellulose.

[106] Lin Z., Guo X., He Z., Liang X., Wang M., Jin G. (2020). Thermal Degradation Kinetics Study of Molten Polylactide Based on Raman Spectroscopy. Polym. Eng. Sci..

[107] Badr Y.A., El-Kader K.M.A., Khafagy R.M. (2004). Raman Spectroscopic Study of CdS, PVA Composite Films. J. Appl. Polym. Sci..

[108] Hu C., Chen X., Chen J., Zhang W., Zhang M.Q. (2012). Observation of Mutual Diffusion of Macromolecules in PS/PMMA Binary Films by Confocal Raman Microscopy. Soft Matter.

[109] Crosby A.J., Lee J. (2007). Polymer Nanocomposites: The “Nano” Effect on Mechanical Properties. Polym. Rev..

[110] Nguyen T.A., Nguyen T.H., Nguyen T.V., Thai H., Shi X. (2016). Effect of Nanoparticles on the Thermal and Mechanical Properties of Epoxy Coatings. J. Nanosci. Nanotechnol..

[111] Navarro Oliva F.S., Sahihi M., Lenglet L., Ospina A., Guenin E., Jaramillo-Botero A., Goddard W.A., Bedoui F. (2023). Nanoparticle Size and Surface Chemistry Effects on Mechanical and Physical Properties of Nano-Reinforced Polymers: The Case of PVDF-Fe_3_O_4_ Nano-Composites. Polym. Test..

[112] Zhang H., Zhu H., Xu C., Li Y., Liu Q., Wang S., Yan S. (2022). Effect of Nanoparticle Size on the Mechanical Properties of Polymer Nanocomposites. Polymer.

[113] Chang A., Babhadiashar N., Barrett-Catton E., Asuri P. (2020). Role of Nanoparticle–Polymer Interactions on the Development of Double-Network Hydrogel Nanocomposites with High Mechanical Strength. Polymers.

[114] Al-Maharma A.Y., Patil S.P., Markert B. (2020). Effects of Porosity on the Mechanical Properties of Additively Manufactured Components: A Critical Review. Mater. Res. Express.

[115] Amza C.G., Zapciu A., Constantin G., Baciu F., Vasile M.I. (2021). Enhancing Mechanical Properties of Polymer 3D Printed Parts. Polymers.

[116] Chaudhary B., Li H., Matos H. (2023). Long-Term Mechanical Performance of 3D Printed Thermoplastics in Seawater Environments. Results Mater..

[117] Vidakis N., Michailidis N., Papadakis V., Argyros A., Spiridaki M., Mountakis N., Nasikas N.K., Petousis M., Kymakis E. (2024). Industrially Scalable Reactive Melt Mixing of Polypropylene/Silver Nitrate/Polyethylene Glycol Nanocomposite Filaments: Antibacterial, Thermal, Rheological, and Engineering Response in MEX 3D-Printing. Mater. Des..

[118] Stern T., Marom G. (2024). Fracture Mechanisms and Toughness in Polymer Nanocomposites: A Brief Review. J. Compos. Sci..

[119] Chen Q., Gong S., Moll J., Zhao D., Kumar S.K., Colby R.H. (2015). Mechanical Reinforcement of Polymer Nanocomposites from Percolation of a Nanoparticle Network. ACS Macro Lett..

[120] Jamil H., Faizan M., Adeel M., Jesionowski T., Boczkaj G., Balčiūnaitė A. (2024). Recent Advances in Polymer Nanocomposites: Unveiling the Frontier of Shape Memory and Self-Healing Properties—A Comprehensive Review. Molecules.

[121] Hiremath A., Murthy A.A., Thipperudrappa S., K N B. (2021). Nanoparticles Filled Polymer Nanocomposites: A Technological Review. Cogent Eng..

[122] Alhavaz A., Rezaei Dastjerdi M., Ghasemi A., Ghasemi A., Alizadeh Sahraei A. (2017). Effect of Untreated Zirconium Oxide Nanofiller on the Flexural Strength and Surface Hardness of Autopolymerized Interim Fixed Restoration Resins. J. Esthet. Restor. Dent..

[123] Acierno D., Patti A. (2023). Fused Deposition Modelling (FDM) of Thermoplastic-Based Filaments: Process and Rheological Properties—An Overview. Materials.

[124] Patti A., Acierno S., Cicala G., Acierno D. (2023). Predicting the Printability of Poly(Lactide) Acid Filaments in Fused Deposition Modeling (FDM) Technology: Rheological Measurements and Experimental Evidence. ChemEngineering.

[125] Chee E., Brown A.C. (2020). Biomimetic Antimicrobial Material Strategies for Combating Antibiotic Resistant Bacteria. Biomater. Sci..

[126] Kyung J.W., Cheong K.H., Woo K.K., Sook S., Hyun K.S., Ho P.Y. (2008). Antibacterial Activity and Mechanism of Action of the Silver Ion in *Staphylococcus aureus* and *Escherichia coli*. Appl. Environ. Microbiol..

[127] Wang L., Hu C., Shao L. (2017). The Antimicrobial Activity of Nanoparticles: Present Situation and Prospects for the Future. Int. J. Nanomed..

[128] Yen L.-T., Weng C.-H., Than N.A.T., Tzeng J.-H., Jacobson A.R., Iamsaard K., Dang V.D., Lin Y.-T. (2022). Mode of Inactivation of *Staphylococcus aureus* and *Escherichia coli* by Heated Oyster-Shell Powder. Chem. Eng. J..

[129] Payne M.P., Shillaker R.O., Wilson A.J. (1993). Phosphoric Acid, Phosphorus Pentoxide, Phosphorus Oxychloride, Phosphorus Pentachloride, Phosphorus Pentasulphide.

[130] Hemmilä M., Hihkiö M., Kasanen J.-P., Turunen M., Järvelä M., Suhonen S., Pasanen A.-L., Norppa H. (2010). Cytotoxicity and Genotoxicity in Vitro and Irritation Potency in Vivo of Two Red Phosphorus-Based Pyrotechnic Smokes. Mutat. Res./Genet. Toxicol. Environ. Mutagen..

[131] Akter M., Sikder M.T., Rahman M.M., Ullah A.K.M.A., Hossain K.F.B., Banik S., Hosokawa T., Saito T., Kurasaki M. (2018). A Systematic Review on Silver Nanoparticles-Induced Cytotoxicity: Physicochemical Properties and Perspectives. J. Adv. Res..

