# Supplementary material for: Printability and Performance Metrics of New-Generation Multifunctional PMMA/Antibacterial Blend Nanocomposites in MEX Additive Manufacturing
Source: Polymers (Basel). 2025 Feb 4;17(3):410. doi: 10.3390/polym17030410 (PMC11820571; doi:10.3390/polym17030410)
Supplement: Supplementary file 1 [file polymers-17-00410-s001.zip › polymers-3467913-SI.pdf]

polymers

MDPI

Supplementary data for article

Printability and Performance Metrics of New-Generation Multifunctional PMMA/Antibacterial Blend Nanocomposites in MEX Additive Manufacturing

Markos Petousis [1], Nektarios K. Nasikas [2], Vassilis Papadakis [3,4], Ioannis Valsamos [1], Katerina Gkagkanatsiou [1], Nikolaos Mountakis [1], Apostolos Argyros [5,6,*], Evgenia Dimitriou [5,6], Nikolaos Michailidis [5,6] and Nectarios Vidakis [1,*]

[1] Department of Mechanical Engineering, Hellenic Mediterranean University, 71410 Heraklion, Greece; markospetousis@hmu.gr (M.P.); valsamos@hmu.gr (I.V.); gkagka@hmu.gr (K.G.); mountakis@hmu.gr (N.M.)
[2] Division of Mathematics and Engineering Sciences, Department of Military Sciences, Hellenic Army Academy, 16673 Attica, Greece; nasikas@sse.gr
[3] Institute of Electronic Structure and Laser of the Foundation for Research and Technology-Hellas (IESL-FORTH)—Hellas, N. Plastira 100 m, 70013 Heraklion, Greece; v.papadakis@uniwa.gr
[4] Department of Industrial Design and Production Engineering, University of West Attica, 12243 Athens, Greece
[5] Physical Metallurgy Laboratory, Mechanical Engineering Department, School of Engineering, Aristotle University of Thessaloniki, 54124 Thessaloniki, Greece; evgeniaod@auth.gr (E.D.); nmichail@auth.gr (N.M.)
[6] Centre for Research & Development of Advanced Materials (CERDAM), Centre for Interdisciplinary Research and Innovation, Balkan Centre, Building B', 10th km Thessaloniki-Thermi Road, 57001 Thessaloniki, Greece
* Correspondence: aargyros@auth.gr (A.A.); vidakis@hmu.gr (N.V.); Tel.: +302-810-379227 (N.V.)

Academic Editor: Javier González-Benito

Received: 23 January 2025
Revised: 31 January 2025
Accepted: 3 February 2025
Published: 4 February 2025

Citation: Petousis, M.; Nasikas, N.K.; Papadakis, V.; Valsamos, I.; Gkagkanatsiou, K.; Mountakis, N.; Argyros, A.; Dimitriou, E.; Michailidis, N.; Vidakis, N. Printability and Performance Metrics of New-Generation Multifunctional PMMA/Antibacterial Blend Nanocomposites in MEX Additive Manufacturing. *Polymers* **2025**, *17*, 410. https://doi.org/10.3390/polym17030410

Copyright: © 2025 by the authors. Licensee MDPI, Basel, Switzerland. This article is an open access article distributed under the terms and conditions of the Creative Commons Attribution (CC BY) license (https://creativecommons.org/licenses/by/4.0/).

Abstract: Poly(methyl methacrylate) (PMMA) is a thermoplastic widely utilized in civilian-, defense-, and medicine-related applications. Therefore, inducing antibacterial properties is an additional asset when infection control is prioritized. To counter this, PMMA was mixed, for the first time, with antibacterial agents (antibacterial blend nanopowder, AP) to curb bacterial proliferation and therefore reduce the chances of infection. The reinforcing efficacy of the blend in PMMA was also assessed. Nanocomposites were developed with various nanopowder concentrations for 3D printing material extrusion (MEX). PMMA/AP nanocomposites were evaluated for their mechanical and rheological properties, thermal stability, morphological, structural, and chemical characteristics, and bacterial resistance (against *Staphylococcus aureus* and *Escherichia coli* (*E. Coli*) using the well diffusion method). The effect on quality metrics, such as the geometrical accuracy and pores of the 3D-printed structure was examined with micro-computed tomography. The modified PMMA had improved properties, such as increased tensile (~20% increase at 2 wt.%) and flexural strength (~10.8% at 4 wt.%), while also having strong antibacterial properties against Staphylococcus aureus and mild antibacterial properties against *E. Coli*. Such improvements add to the expanding portfolio of biomaterials, such as their use in the demanding defense sector and the medical field.

Keywords: poly(methyl methacrylate) (PMMA); material extrusion (MEX); additive manufacturing; nanocomposites; mechanical characterization; *Staphylococcus aureus*; *Escherichia coli*

S.1. Mechanical tests graphs

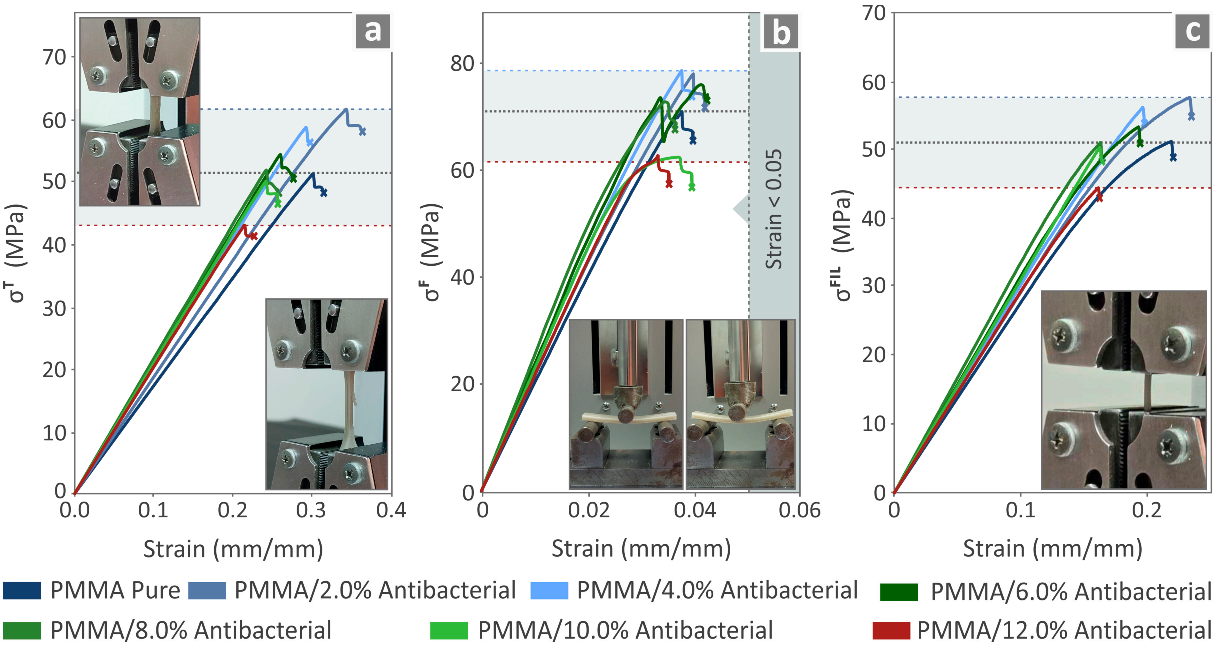

Figure S1. For all the PMMA/antibacterial blend compounds tested (a) Tensile stress vs. strain curve, (b) Flexural stress vs. strain curve, and (c) Filament tensile stress vs. strain curve.

S.2. 3D printing settings and samples' geometry in accordance with the standards

Parameters	Value	Units
PMMA Antib. content	0.0–2.0–4.0–6.0–8.0–10.0–12.0	%
Printing orientation	±45	deg
Nozzle temperature	270	°C
Bed temperature	100	°C
Layer thickness	0.2	mm
Number of perimeters	2	–
Fill density	100	%
Travel speed	40	mm/sec

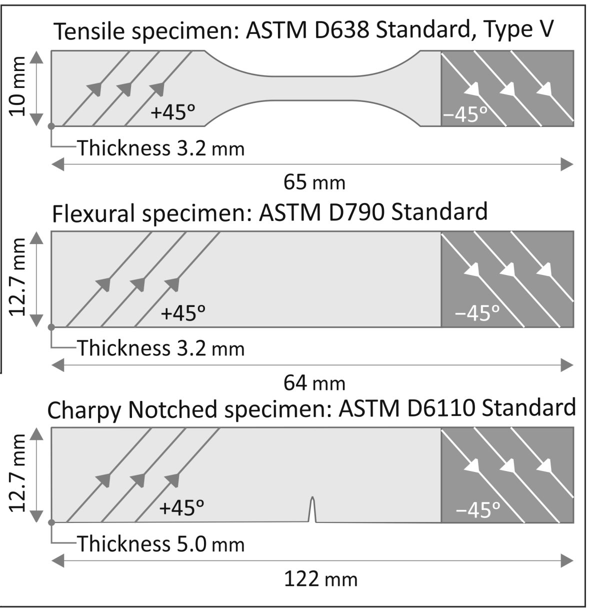

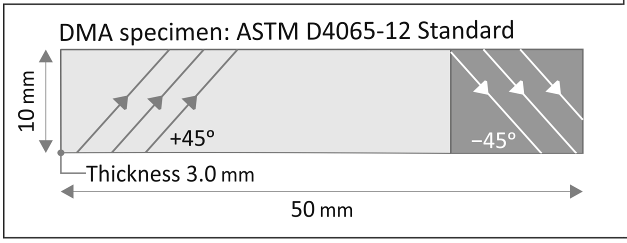

Figure S2. Settings used for parts manufacturing with MEX 3D printing and the geometry of the samples manufactured, following the respective standards. The infill pattern of the 3D printing structure is also indicated.

S.3. Summary of the experimental findings

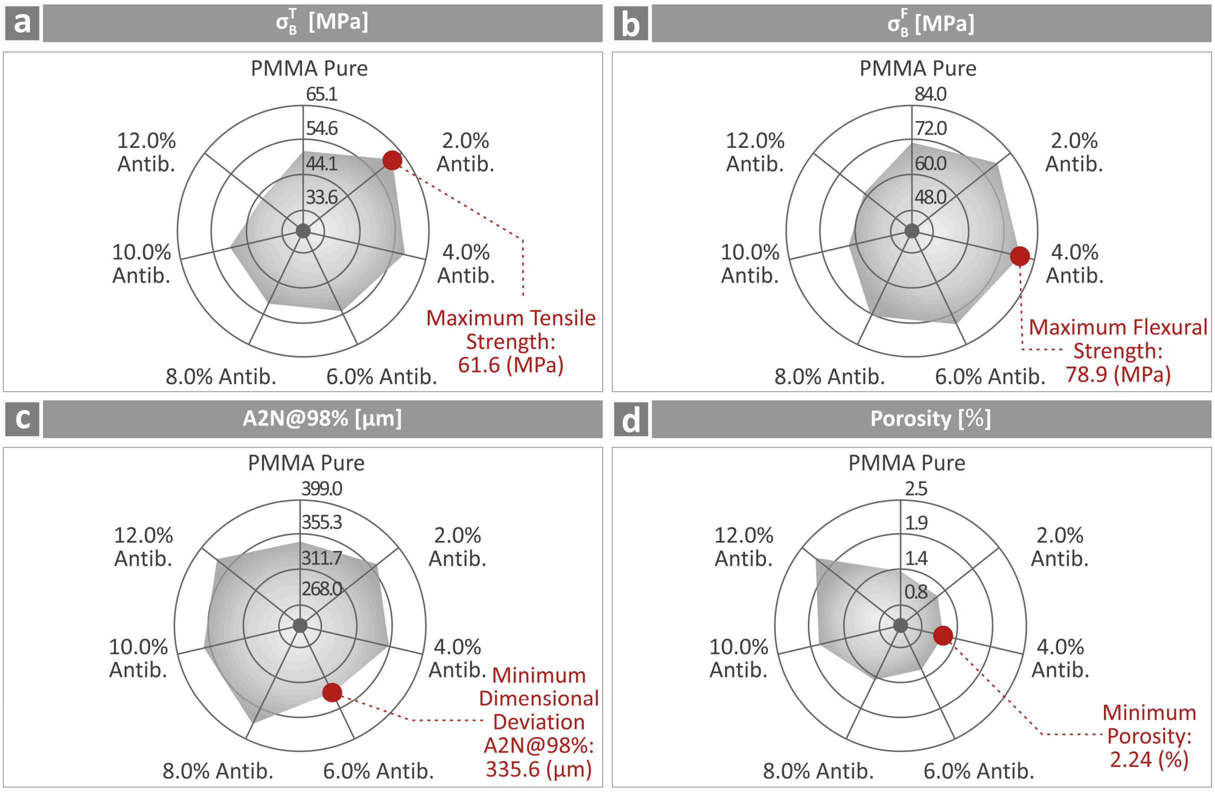

Figure S3. Spider graph summarizing the main experimental findings. The highest values are indicated.

S.4. Raman spectroscopy

The powerful and non-destructive tool of Raman spectroscopy was utilized in order to investigate the various structural characteristics of the prepared nanocomposites. For this purpose, we utilized a micro-Raman setup, the Labram HR-Horiba Scientific. The laser line was produced from a continuous wave (cw) solid-state laser operating at 532 nm wavelength with an output of 90 mW. The focus of the laser line onto the sample was achieved by a 50x microscope lens and the backscattered light was collected through the same optical path. The working distance of the microscope lens was set at 10.6 mm. The numerical aperture was adjusted to 0.5 (LMPlanFL-N). Aiming to avoid any decomposition or alteration to the samples under investigation by the intense focused laser beam we reduced the laser's power by employing a Neutral Density (ND) filter with transmittance of 5%, to achieve 2 mW power, onto the sample's surface. In the lateral direction, the laser spot was characterized by a value of 1.7 μm. In the axial direction, it was set at 2.0 μm. The resolution of the Raman spectra was adjusted at 2.0 cm^{-1}. This was achieved by using a grating having 600 grooves/mm. The range of the spectra for the acquisition of all spectra signatures origination from every sample extended from 50 to 3900 cm^{-1} and was covered by three (3) optical windows. The time for acquisition was adjusted to 10 s and the accumulative spectra were adjusted to five (5) ensuring a vibrational spectrum of high-quality in terms of signal-to-noise ratio.

In the following Table S1 the related Raman peaks from the PMMA pure sample are presented as extracted from the literature together with their reference.

Table S1. Significant Raman peaks and their related assignments from PMMA pure.

Wavenumber (cm^{-1})	Intensity	Raman peak assignment
598	Strong	C-COO vibration, C-C-O symmetric vibration [1]
734	Small	C-H out-of-plane bending [2,3]

811	Strong	CH2 vibration [1]
844	Small	Phenyl ring vibration [4]
911	Small	C-H in-plane bending [3]
964	Strong	O-CH3 rocking [1]
985	Strong	C-C and C-O vibration [5]
1120	Medium	Skeletal vibrations, C–C bonds [1,5]
1181	Small	Skeletal vibrations, C–O– C, C-COO bonds [1,2,5]
1240	Small	C-O-C stretching [3]
1326	Small	C-O-C stretching [3]
1449	Strong	C-H_3 deformation [1,3]; C-H_2 deformation [2,3]; C-H_3 symmetric bending [3,4,6];
1727	Strong	C = O bond [1,7] C-O-C symmetric stretching [8]
2843	Medium	O-CH_3 vibration [1]
2951	Strong	CH_2 and C-H asymmetric stretching [1,5]
3000	Medium	C-H stretching [3]

References

1. Veluthandath, A. V; Bisht, P.B. Identification of Whispering Gallery Mode (WGM) Coupled Photoluminescence and Raman Modes in Complex Spectra of MoS2 in Polymethyl Methacrylate (PMMA) Microspheres. *J Lumin* 2017, *187*, 255–259, doi:https://doi.org/10.1016/j.jlumin.2017.03.031.
2. Zimmerer, C.; Matulaitiene, I.; Niaura, G.; Reuter, U.; Janke, A.; Boldt, R.; Sablinskas, V.; Steiner, G. Nondestructive Characterization of the Polycarbonate - Octadecylamine Interface by Surface Enhanced Raman Spectroscopy. *Polym. Test.* 2019, *73*, 152–158, doi:https://doi.org/10.1016/j.polymertesting.2018.11.023.
3. Stuart, B.H. Temperature Studies of Polycarbonate Using Fourier Transform Raman Spectroscopy. *Polymer Bulletin* 1996, *36*, 341–346, doi:10.1007/BF00319235.
4. Resta, V.; Quarta, G.; Lomascolo, M.; Maruccio, L.; Calcagnile, L. Raman and Photoluminescence Spectroscopy of Polycarbonate Matrices Irradiated with Different Energy 28Si+ Ions. *Vacuum* 2015, *116*, 82–89, doi:https://doi.org/10.1016/j.vacuum.2015.03.005.
5. Makarem, M.; Lee, C.M.; Kafle, K.; Huang, S.; Chae, I.; Yang, H.; Kubicki, J.D.; Kim, S.H. Probing Cellulose Structures with Vibrational Spectroscopy. *Cellulose* 2019, *26*, 35–79, doi:10.1007/s10570-018-2199-z.
6. Lin, Z.; Guo, X.; He, Z.; Liang, X.; Wang, M.; Jin, G.; Lin, Z. N., X. M. Guo, Z. P. He, X. R. Liang, M.M.W. and G.J.; Lin, Z.; Guo, X.; He, Z.; et al. Thermal Degradation Kinetics Study of Molten Polylactide Based on Raman Spectroscopy. *Polym Eng Sci* 2021, *61*, 201–210, doi:https://doi.org/10.1002/pen.25568.
7. Badr, Y.A.; El-Kader, K.M.A.; Khafagy, R.M. Raman Spectroscopic Study of CdS, PVA Composite Films. *J Appl Polym Sci* 2004, *92*, 1984–1992, doi:10.1002/app.20017.
8. Hu, C.; Chen, X.; Chen, J.; Zhang, W.; Zhang, M.Q. Observation of Mutual Diffusion of Macromolecules in PS/PMMA Binary Films by Confocal Raman Microscopy. *Soft Matter* 2012, *8*, 4780–4787, doi:10.1039/C2SM07299H.

Disclaimer/Publisher's Note: The statements, opinions and data contained in all publications are solely those of the individual author(s) and contributor(s) and not of MDPI and/or the editor(s). MDPI and/or the editor(s) disclaim responsibility for any injury to people or property resulting from any ideas, methods, instructions or products referred to in the content.
